# How subcultures emerge

**DOI:** 10.1017/ehs.2023.19

**Published:** 2023-07-12

**Authors:** Petr Tureček, Michal Kozák, Jakub Slavík

**Affiliations:** 1 Department of Philosophy and History of Science, Faculty of Science, Charles University, Prague 2, 128 00, Czech Republic; 2 Center for Theoretical Study, Charles University and Czech Academy of Sciences, Jilská 1, Prague 1, 110 00, Czech Republic; 3 Department of Mathematics, Faculty of Nuclear Sciences and Physical Engineering, Czech Technical University in Prague, Trojanova 13, 120 00, Prague 2, Czech Republic; 4 Institute of Information Theory and Automation, The Czech Academy of Sciences, Pod Vodárenskou věží 4, 180 00, Prague 8, Czech Republic

**Keywords:** cultural evolution, cultural divergence, sympatric speciation, Galton–Pearson model, PVDI

## Abstract

Sympatric speciation is typically presented as a rare phenomenon, but urban subcultures frequently emerge even in the absence of geographic isolation. Is there perhaps something that culture has but biological inheritance does not that would account for this difference? We present a novel model that combines assortative interaction and multidimensional inheritance. Our computer simulations show that assortment alone can lead to the formation of cohesive clusters of individuals with low within-group and large between-group variability even in the absence of a spatial separation or disruptive natural selection. All it takes is a proportionality between the variance of inputs (cultural ‘parents’) and outputs (cultural ‘offspring’). We argue that variability-dependent inheritance cannot be easily accomplished by genes alone, but it may be the norm, not the exception, in the transmission of culture between humans. This model explains the frequent emergence of subcultures and behavioural clustering in our species and possibly also other cultural animals.

## Graphical abstract

**Figure:**
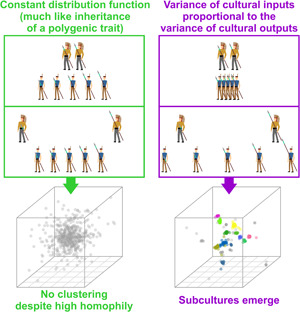

**Social media summary:** New model shows how subcultures, unlike species, pop-up from preferences to interact with self-similar individuals.

## 1. Introduction

There is a long-standing debate about the possibility of sympatric speciation, i.e. the emergence of multiple species from one without geographic isolation (Coyne & Orr, 2004; Dieckmann & Doebeli, 1999; Gavrilets, 2014; Kondrashov & Kondrashov, 1999; Mayr, 1947; Nosil, 2008; Smith, 1966; Via, 2001). Although the importance of the geographical aspect of speciation has lately been questioned (Hubert, Calcagno, Etienne, & Mouquet, 2015; Schluter, 2009), spatial isolation is still believed to play a pivotal role in divergent evolution because it limits the gene flow between adjacent populations (Chakraborty & Nei, 1982; Mallet, Meyer, Nosil, & Feder, 2009). The conundrum of the emergence of distinct groups of organisms, coined as the ‘mystery of mysteries’ by Charles Darwin, was not originally reserved just for the formation of species (Mank, 2009). It referred to the unfolding of ruptures in the continuum between groups with high between-group and low within-group variation (Pavlinov, 2013), because Darwin stressed the absence of fundamental differences between varieties, subspecies, species and higher taxa (Darwin, 1859). Differences between such categories, e.g. between varieties and species, were supposed to be a question of scale rather than quality. Current research tends to support this view (Ereshefsky, 2010; Schluter, 2009). It seems that reproductive isolation can emerge quite easily even under conditions characterised by an uninterrupted gene flow (Nosil, 2008). Two distinct varieties may develop into two separate species via several broadly overlapping stages: (1) a homogeneous gene flow between populations; (2) a heterogeneous gene flow where the alleles directly linked to diverging traits rarely cross boundaries between populations; and finally (3) separate species that meet the condition of full reproductive isolation (Roux et al., 2016). It should be noted, however, that resistance from the mainstream against this view is still quite considerable (Mallet et al., 2009).

Nevertheless, the whole subject of structured biota is no less challenging than its best-known species-oriented incarnation (Pavlinov, 2013). Why do, for example, distinct cultures, subcultures or ethnic groups exist? To find an answer, we must clarify what we mean by ‘subculture’. We use this term because we focus on models from the field of cultural evolution. Strictly speaking, there is no formal difference between a ‘subculture’ as we use the term in this study and a morphologically defined species (Claridge, Dawah, & Wilson, 1997). A subunit within a higher-order (structured) biota is a cluster of individuals in a trait space distinguished from other similar clusters by a gap in the continuum of transitive forms. Modern clustering algorithms, such as the HDBSCAN (Hierarchical Density-Based Spatial Clustering of Applications with Noise; Campello, Moulavi, & Sander, 2013), are designed to detect precisely that: distinct groups of observations separated by gaps, not arbitrary chunks of elongated clusters or continuous clines (the usual outcome of *K*-means clustering and many other popular methods; Pedregosa et al. (2022); see also Rosenberg et al. (2005) for a detailed discussion of clines vs. clusters in the context of human populations). Our definition of subculture is therefore a rather pragmatic one: when multiple distinct clusters in a trait space (culture space) are detected, we call them subcultures. If clustering does not occur we conclude that subcultures are absent.

One of the cornerstones of divergence and speciation is a positive assortment, that is, an increased likelihood of interaction between mutually similar individuals (Gavrilets, 2004; Jokinen et al., 2017). Straightforward ‘phenotype matching’ is a more potent diversification driver than parental imprinting, and both these processes stand in contrast to the homogenising effect of oblique imprinting, in which case individuals form preferences on the basis of the whole adult population and any correlation between their own and preferred partner's phenotype is absent (Verzijden, Lachlan, & Servedio, 2005). Both assortative social learning and mating seem almost omnipresent in human populations (Luo, 2017) and have been abundantly documented also in non-human animals (Huber, De León, Hendry, Bermingham, & Podos, 2007; Jiang, Bolnick, & Kirkpatrick, 2013). They have been previously identified as a necessary but not sufficient condition of sympatric speciation (Dieckmann & Doebeli, 1999).

Many authors have verbalised the intuition that social learning and imprinting must play an important role in bird speciation since songbirds are responsible for roughly 60% of all bird species diversity and they also represent the clade that relies on cultural transmission the most (Nottebohm, 1972; Vaneechoutte, 1997) Yet standard models that represent culture in a particulate ‘memetic’ manner suggest that a situation, where a behaviour under sexual selection is fixed genetically, favours the diversification and speciation more than if the behaviour is socially transmitted (Olofsson, Frame, & Servedio, 2011; Olofsson & Servedio, 2008).

We propose that the key difference between biological inheritance and cultural transmission may lie in the probability density function which approximates the generation of a new trait value (of an offspring or learner) from multiple predecessors (parents or role models).

### 1.1 Non-particulate inheritance models

Besides models that assume discrete cultural replicators (Axelrod, 1997; Creanza & Feldman, 2014; Gavrilets, 2004; Mcelreath, Boyd, & Richerson, 2003; Olofsson et al., 2011) and Henrich's uniparental model, where a single, most successful individual is imitated by everyone else (Henrich, 2004), two models of continuous trait inheritance have recently been employed in studies of cultural evolution (Cavalli-Sforza & Feldman, 1981; Tureček, Slavík, Kozák, & Havlíček, 2019). These models do not rely on – in this context restrictive – beliefs about additional genetic variation. Rather, they can be viewed as general inheritance models such as those which preceded or existed in parallel with Mendelism. They have been overlooked owing to evolutionary biologists’ focus on genetic models which are believed to provide a good approximation of the elementary form of organismal inheritance.

Both continuous probabilistic models are based on simple blending inheritance but replace the problematic assumption of a trait of the offspring (*t*_*o*_) being exactly in the arithmetic mean of parental values 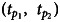 by the assumption of a random normal distribution around the arithmetic mean.

The Galton–Pearson (GP) model, used previously to approximate continuous cultural inheritance, assumes a “mutation term” characterised by a constant standard deviation (*η*) independent of the difference between parental values. In symbols, we have1
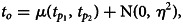
where
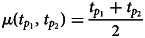
denotes the arithmetic mean and N(*m*, *V*) is a normal distribution with mean *m* and variance *V*. The model in Eqn (1) can be generalised to account for multiple (say *M*) parents using
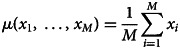
instead. However, for the sake of clarity, we will further discuss only the variant with two parents. This model is based on Galton's experiments with inheritance of the seed size in sweat pea and investigations of heredity of human height (Stanton, 2001). It can very well approximate genetic inheritance if we assume a large population and a high number of freely recombining genes with additive genetic variance (Fisher, 1918). Despite its genetic roots, it has been used in approximations of cultural inheritance of continuous traits (Cavalli-Sforza & Feldman, 1976, 1978, 1981).

The GP model assumes that a pair of highly diverse parents and a pair of identical parents have the same distribution of offspring if they have equal mean trait values. This assumption is acceptable when a large number of freely recombining elements, such as genes, execute the inheritance for their bearers. It is, however, quite unrealistic when individual agents directly observe and imitate the phenotypes of multiple role models. It is so for two reasons: (1) it is more difficult to intuitively assess the average of two values when they are further apart; and (2) when two successful individuals differ more, it is safe to assume that the span of acceptable trait values is wider.

Imagine a young seamstress who wants to fit into a town she has just moved to. She approaches two prestigious individuals, perhaps older seamstresses, to see about the length of skirts that people tend to buy around there. She soon discovers that each of her role models makes quite different skirts. One makes short skirts, while the other makes rather long ones. Eventually, the new seamstress opts for a practical skirt length somewhere in between. Then another seamstress approaches the same two older, well-established, seamstresses and develops a strategy inspired by both but closer to the one with the short-skirt strategy. When multiple youngsters learn from the same pair of elders, we can describe the distribution of their resulting skirt lengths by a bell curve around the mean of the two ‘cultural parents’.

The GP model implicitly assumes that the distribution of young seamstresses’ strategies would remain unchanged even if the two master seamstresses produced and recommended identical skirts of average length: there would be an identical mean to imitate but no variance. There is no reason to assume that the apprentice seamstresses would use the information on mean acceptable trait value but remain oblivious to the range of acceptable trait values.

The parental variability-dependent inheritance (PVDI), suggested as an alternative to the GP model (Tureček et al., 2019), supposes that the standard deviation of offspring is proportional (with ratio *ν*) to the standard deviation of parental trait values. In biparental inheritance, this is equal to one-half of the parental distance. In symbols, we write2
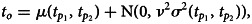
where
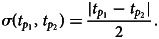


Similarly to above, one could generalise the formula to *M* parents by considering
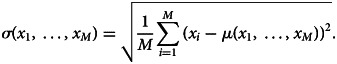


In the PVDI system, homogeneous parents (or cultural parents) produce homogeneous offspring (cultural offspring), while heterogeneous parents produce heterogeneous offspring.

Despite a rich discussion between modellers that showed that discrete cultural replicators may emerge from continuous transmission dynamics (Henrich & Boyd, 2002) and their opponents who claimed that assumptions leading to such extreme scenarios are unrealistic (Claidière & Sperber, 2007), little attention has been paid to the cognitive realism of a constant error term in cultural transmission. PVDI does not require agents to have an inherent source of constant cultural ‘noise’. Rather, it assumes that the deviation is relative to the observed sources of culturally transmitted information. This approach is in line with works on conformity and distinctiveness, where agents strive to be, for instance, 1 standard deviation left from the mean on the left-right political spectrum, rather than ‘15 ideological units’ from the mean regardless of the distribution of other agents (Smaldino & Epstein, 2015). Moreover, in contrast to such relativistic models, PVDI does not require agents to perceive and assess the whole population of conspecific at once. We believe that many classical models might benefit from the introduction of variance-dependent terms.

**Figure 1.**
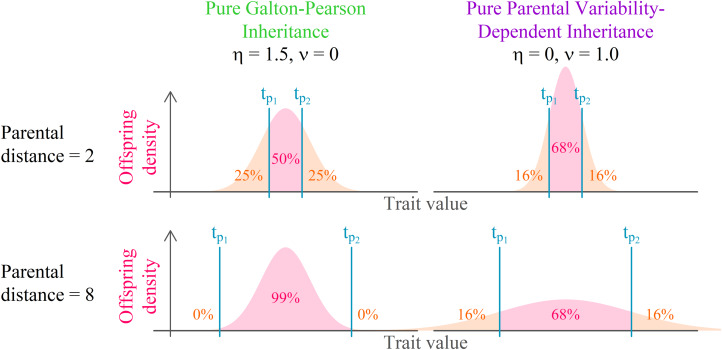
Model comparison. Offspring distribution function is given by the arithmetic mean of parental values and phenotypic mutation, see Eqns (1) and (2). In a system with pure parental variability-dependent inheritance (PVDI), the proportion of offspring between parental values is constant. In a system with Galton–Pearson (GP) inheritance, the proportion depends on the distance between parental values.

Both non-particulate models from Eqns (1) and (2) can be easily combined and generalised to a multidimensional form,3
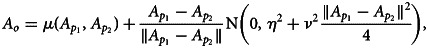
where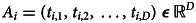 represents an individual's position in a Euclidean *D*-dimensional trait space (see Supplement S1 for a detailed derivation). There, the offspring assumes a position on a vector connecting her parents. Her distance from the point in-between parental positions is normally distributed. The lower the coefficient of proportional variability (*ν*) relative to the constant offspring variation (*η*), the closer the model is to a pure Galton–Pearson inheritance. A visual comparison of the models is shown in Figure 1. Vectorising the inheritance function deals with previous criticism that older models of cultural transmission assume independence of every cultural feature of other cultural features (Axelrod, 1997).

## 2. Methods

### 2.1. Overview

We employ an agent-based simulation to study the emergence of a structured biota under the assumption of assortative pairing. Individuals close to each other in a multidimensional trait space are considered similar. We assume no external natural selection acting on the population and only relative preferences for pairing between individuals. In our simulations, relative preferences between agents are determined by a homophily coefficient (*h*) expressing a preference for self-similarity. If *h* is negative, we observe a negative assortment where dissimilar individuals are more likely to pair. When *h* is positive, there is a positive assortment and similar individuals pair with a higher probability. For *h* = 0, there is no assortment, and the pairing is completely random.

In each step, each agent selects an interaction partner (‘role model’) and modifies her position along a vector between her and her interaction partner. The value 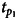 in Figure 1, can be in this context interpreted as the agent's original position, 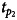 as the selected role model's position, and offspring density as the agent's resulting position distribution after the interaction.

We use the continuous inheritance model, with PVDI and GP terms combined, since a pure PVDI model implies that if cultural parents share a trait value, the offspring become perfect learners who cannot make errors. The standard deviation in the combined model is controlled by two parameters, constant offspring variation *η* and coefficient of proportional variability *ν*, that are introduced in the description of continuous cultural inheritance models above.

We offer this narration for the combined model: agent preferences are centred around a mean of their cultural parents and their standard deviation is proportional to the standard deviation of the cultural parents: N(*μ*(*t*_*p*_), *ν*^2^*σ*^2^(*t*_*p*_)), where the variance of the distribution is just the standard deviation squared. Because an agent is not always able to match its preferences exactly, the resulting position is the sum of the preferred position and some random influence independent of parental traits: N(0, *η*^2^). The additivity of variance (Fisher, 1918), N(*μ*(*t*_*p*_), *ν*^2^*σ*^2^(*t*_*p*_)) + N(0, *η*^2^)  ≈  N(*μ*(*t*_*p*_), *ν*^2^*σ*^2^(*t*_*p*_) + *η*^2^), allows us to draw just one random number per modification of cultural position and yet consider both sources of randomness in cultural inheritance simultaneously (see Supplement 1 for details). With the probability proportional to the pink area in Figure 1, the agent will end up between its original position and the role model's position. For cases where *η* = 0, this probability *p*_*between*_ can be calculated from *ν* using
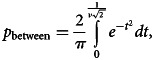
which is derived from the normal cumulative distribution function (see Supplement S2 for details). Because normal distribution is symmetric, the agent extremises its own position away from the role model with the same probability as it overshoots the role model's position along the vector that connects them, therefore
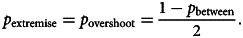


For *η* > 0 these formulas would depend on the distance between the agents. However, for pure PVDI, we can calculate that e.g. *p*_between_ = 0.68 for *ν* = 1, and *p*_between_ = 0.38 for *ν* = 2, or for many regularly spaced values of *ν* we find that *p*_between_ = 0.5 when *ν* = 1.48 or that *p*_between_ = *p*_extremise_ = *p*_overshoot_ when *ν* = 2.32. (The values of *ν* were obtained using numerical methods and rounded to 2 decimal places. Analytical solution is not possible, because the Gaussian error function has no closed-form expression.) On a similar note, it is possible to run a series of simplified simulations and find out that for *ν* ≥ 2.10 two points that serve as role models to each other are more likely to end up further apart after the cultural exchange than they were before it.

While the example with skirt length (or canoe size, spear length, ratio of red and white cattle, age at marriage, amount of milk to put in coffee, etc.) represents a special unidimensional case of our model, we focus on cultural transmission or procreation in a multidimensional trait space. We do not need to suppose that in reality, the trait space that is used to represent differences and similarities between individuals has such a straightforward one-to-one correspondence with the design-space of an artefact (Mesoudi & O'Brien, 2008). The model bears a strong resemblance to the idea of a broad underlying culture space described in the work of Sperber (1996). It represents a useful tool that generalises to discrete cultural variants if the probability (continuous) that certain behaviour is executed becomes the centre of the analysis instead of the behaviour's single occurrence (binary).

### 2.2. The formal model

In each simulation run, each agent *A*_*i*_ in a population *P* of size *n* is represented by its position in a Euclidian *D*-dimensional space (a ‘trait space’ or ‘culture space’):4
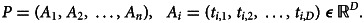


The difference between any two individuals in the trait space can be calculated as the distance between two points in a *D*-dimensional space:5
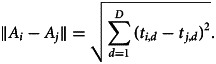


In this trait space, we are simulating a process of non-particulate position inheritance. There is no external natural selection operating on the population and only relative preferences for pairing between individuals. In each time step or ‘generation’ *g*, each agent selects a partner probabilistically. The relative preference of the focal individual *A*_*i*_ for agent *A*_*j*_ is given by6
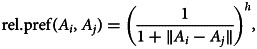
where *h* is a homophily coefficient; a measure of preference for self-similarity. This well-behaved function ensures that self-preference, i.e. a preference for individuals whose distance from an agent is zero, serves as a referential value of 1, and other preferences follow proportionally depending on *h*.

Relative preference of agent *A*_*i*_ for agent *A*_*j*_ divided by the sum of all relative preferences of *A*_*i*_ defines probability7
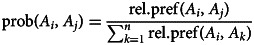
that *A*_*i*_ selects *A*_*j*_ as role model. Role models are sampled with replacement; one agent can therefore serve as the selected role model to more than one agent.

If *h* is negative, we observe heterophily, that is, negative assortative “mating” where dissimilar individuals pair with a higher probability. For a positive *h*, pairing follows homophily, positive assortative “mating”, where similar individuals pair with a higher probability. For *h* = 0, there is no assortment, relative preference for all individuals is 1, and pairing is completely random. The absolute value of homophily |*h*| corresponds to the strength of assortment. For *h* = 1, agent *A*_*j*_ who is twice as close to the focal agent *A*_*i*_ as agent *A*_*k*_, will be selected as a partner by *A*_*i*_ approximately twice as often as *A*_*k*_ (assuming *A*_*i*_ − *A*_*j*_ and *A*_*i*_ − *A*_*k*_ are significantly larger than 1). For *h* = 2, *A*_*j*_ will be selected approximately four times as often as *A*_*k*_. For *h* = 3, *A*_*j*_ will be selected approximately eight times as often as *A*_*k*_, etc. Similarly, for *h* = −1, *A*_*k*_ will be selected twice as often as *A*_*j*_ etc. *mutatis mutandis*.

Because each individual *A*_*i*_(*g*) at time step *g* selects one role model *A*_*j*_(*g*), we obtain *n* pairs of parents in all time steps. Each pair of parents creates one offspring, denoted for this purpose *A*_*i*_(*g* + 1), whose position in the trait space is given by probabilistic non-particulate inheritance combining GP and PVDI terms (see Eqn 3), that is,8
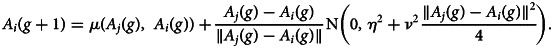


The agent's position in the following *g* + 1 timestep, *A*_*i*_(*g* + 1), therefore always lies on a line connecting the parental points (a variant of the model with additional normal noise can be found in Supplement S7). The distance of *A*_*i*_(*g* + 1) from the arithmetic average of *A*_*i*_(*g*) and *A*_*j*_(*g*) is normally distributed. This approach has the advantage of arriving at identical results under any rotation of coordinate basis.

The construction of agent configuration of a population *P* in *g* + 1, *P*(*g* + 1), from *P*(*g*) takes only a single time step, which means that all agents in our simulation alter their positions in synchrony.

A similar model that pairs individuals exclusively for each time step can also be constructed. With this modification, *n*/2 pairs of agents are formed in generation *g*, each creating two new agents, ensuring that the population size remains constant, whereby the subsequent time step *g* + 1 follows the same inheritance algorithms. This modification invites new interpretations: (1) a vertical cultural transfer from biological parents to biological offspring, meaning that one step is identified with a biological generation; or (2) exclusive interactions, such as discussions, conversations and exchanges of opinions, after which the positions of both interacting individuals change simultaneously. In this model, an additional specification of the algorithm must ensure within-pair exclusivity. The selection of interaction partners in step *g* is decided one agent at a time in a random order. If an agent is already selected as an interaction partner, she does not select a partner of her own and cannot be selected as and interaction partner by any other agent. In any case, both models – let us refer to them as ‘inspirational’ and ‘interactional’ respectively – are highly similar and lead to equivalent conclusions. The ‘inspirational’ model, which is the focus of this paper, does not require any additional specifications.

### 2.3. Computer simulation

The initial state of the computer simulation was set to a single cloud of normally distributed points with standard deviation *σ* = 100 along each trait value. Simulation results were obtained for *D* = 10.

Simulation parameters (*n* = 250, 500, 1000, *η* = 1, 10, 100, 0 ≤ *h* ≤ 4, 0 ≤ *ν* ≤ 3.0) were chosen so as to demonstrate important transitions between systems with different frequencies of divergence. From the initial configuration, the partner selection + position inheritance algorithm was iterated for 200 steps in each simulation run (1000 runs per parameter combination).

### 2.4. Analysis and visualisation

The average distance between agents in the trait space was calculated to assess whether trait space collapses into a single point (variability loss) or enters a feedback loop of ongoing expansion (variability explosion, see Supplement S4, Figure S3). The effective dimensionality of the trait space was quantified by the proportion of variance of agent positions explained by the first three principal components (PCs) of the *D*-dimensional trait space and by the number of PCs necessary for capturing 99% of all variance. These measures were highly correlated (cor = 0.9), so in the following we only worked with the first measure. We chose variance explained by the first three dimensions because it has an intuitive interpretation in the context of the resulting images: what proportion of overall variance in 10 dimensions is captured in the 3D scatterplot. If this proportion is for example 95%, we can conclude that despite the model running in 10 dimensions, the effective dimensionality of the population is lower because we would obtain an almost identical distance matrix capturing differences and similarities between agents if we used only the PC1–PC3 space. The number of distinct clusters was evaluated using the HDBSCAN algorithm (for a description of the algorithm, see Supplement S3), a hierarchical version of DBSCAN (Density-Based Spatial Clustering of Applications with Noise), which is an important part of the rich tradition of density-based clustering methods in biology (Edla & Jana, 2012). Unlike DBSCAN, HDBSCAN sidesteps the problem of how close points should be to be considered a part of the same cluster.

**Figure 2.**
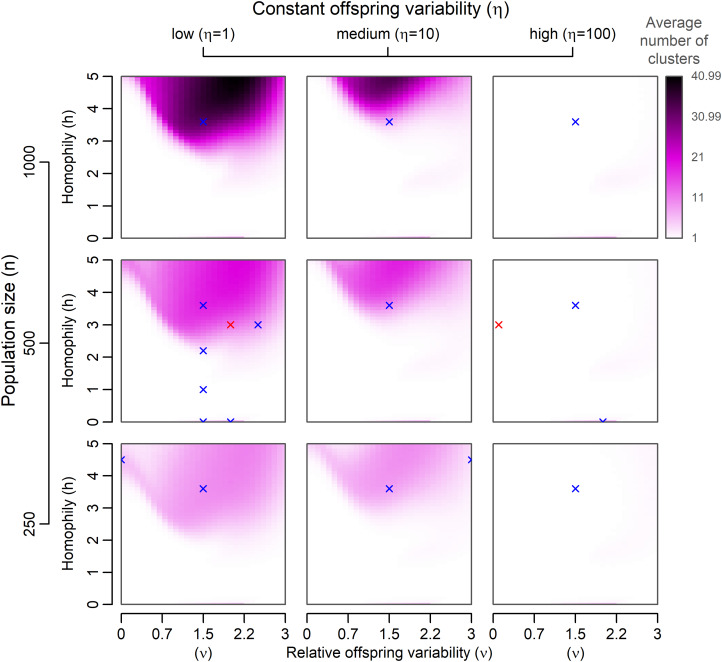
A graphical summary of the number of subcultures after 200 model generations. The points in the 10-dimensional culture space were normally distributed across all dimensions at the beginning of each simulation run, and 1000 simulation runs were executed for each parameter combination. Red crosses indicate the values of parameters used for single-run examples included in the main article. Blue crosses indicate examples available in the Supplementary Material.

The development of agent network in the trait space is visualised across time steps as a series of static images and as an animation capturing the dynamical process of clustering (see Supplementary animations). The 3D scatterplots work with the first three PCs (standardised to have mean = 0 and SD = 1 along each PC) and summarise the layout of points in a *D*-dimensional trait space. To minimise scatterplot rotation between adjacent frames, PC1, PC2 and PC3 are mapped to axes *x*, *y* and *z* through PC rearrangement and reversion, such that cor(*x*_*g*_, *x*_*g*+1_) + cor(*y*_*g*_, *y*_*g*+1_) + cor(*z*_*g*_, *z*_*g*+1_) is maximised. The points indicating agents’ relative positions in PC1–PC3 space are coloured according to their assignment to distinct clusters. The biggest subgroup from cluster Γ in time step *g* that belongs to a single cluster in time step *g* + 1 inherits the colour of Γ from the previous step. The measure of effective dimensionality (Figure S2), the number of distinct clusters (Figure 2), and the average distance between agents (Figure S3) are also visualised as variables dependent on time in a graphical summary of a single simulation run. The proportion of non-clustered noise is assesed at the end of each simulation run (Figure S4).

The average values of measures for all simulation runs with the same parameter configuration are visualised as colour shades depending on parameters *n*, *h*, *ν*, and *η*.

A straightforward extension of the model allows us to focus on relative rather than absolute differences between individuals in processes that drive cultural acquisition. In such a model, we normalise the positions of agents so as to maintain the average distance between agents constant after each step. Thanks to this additional step, the model leads neither to variability loss (where *ν* and *η* are too low) nor to variability explosion (where *ν* is too high), which were both present in the original model. In effect, normalisation binds *η* to the overall population variance while keeping it independent of the two particular values selected as parental traits. This resembles Fisher's genetic model which reconciled biometric inheritance with Mendelism under the assumption of additive genetic variance (Fisher, 1918). In the main paper, we abstained from any extensions, including normalisation, because our aim was to demonstrate the potential of a very simple model, one relying only on bilateral relationships between interacting agents, to sustain a reasonable cultural variation. A more detailed exposition of the normalised model can be found in Supplement S6.

Demonstrative examples were run using R (RCoreTeam, 2019). The code for parallel runs was written in Python (Van Rossum & Drake, 2009) using *Numpy* (Van Der Walt, Colbert, & Varoquaux, 2011) infrastructure and delegated to processor cores using R packages *parallel* (RCoreTeam, 2019) and *reticulate* (Allaire & Ushey, 2020). Visualisations were created using the base R graphics.

## 3. Results

**Figure 3.**
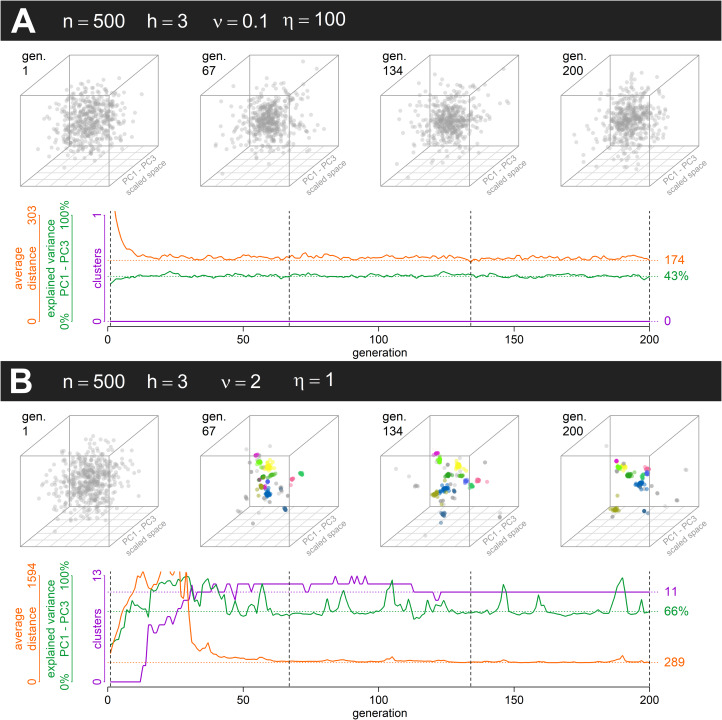
Two simulation runs, one of a system strongly influenced by GP inheritance (a) and one strongly influenced by PVDI (b). A configuration of points across the first three principal components is displayed at the beginning and after each third of the simulation run. The system with PVDI inhabits the culture space in a discontinuous manner and forms distinct clusters which are stable over time. (See also Supplementary animations 3A and 3B; all supplementary animations are deposited in a separate folder at https://doi.org/10.17605/osf.io/pvyhe, see S6B in the Supplement and the corresponding animation for an approximately intermediate case.) Standardised first three principal components (PC1–PC3 scaled space; for further elucidation see the Methods) rotated to minimise changes between adjacent images are used to visualise the 10-dimensional configuration in 3D scatterplots.

We found that distinct groups with a high between-group and low within-group variance emerge from positive assortment alone when PVDI prevails over GP, that is, when constant standard deviation *η* in an inheritance model that combines the GP with PVDI is small (see an example run in Figure 3b). The emergence of distinct subcultures was observed even though we did not introduce any explicit threshold in mutual similarity below which agents tend to coordinate their positions and above which they tend to actively differentiate (Turner & Smaldino, 2018).

In a system with a considerable influence of constant offspring variation (such as *η* = 100), assortment alone cannot lead to the emergence of distinct groups (Figure 2). Instead, the agents form a single uniform cluster (Figure 3a). This outcome is affected neither by homophily nor by population size. In some intermediate cases (for instance supplementary Figure S6C, *n* = 1000, *h* = 3.6, *ν* = 1.5, *η* = 10) we may see clusters forming and later collapsing into a single bulk of points. This scenario is likely when the simulation starts with enough initial variance, relatively low *ν*, and homophily that allows occasional selection of role models from clusters other than the agent's own. This combination tends to bring clusters closer together. With narrowing the gaps between clusters, higher constant *η* starts playing a significant role and further blurs the distinctions. Because there is higher chance of agent bridging two existing clusters by ending up between them in large populations, the domain where clusters are present at generation 200 shifts upwards with the increase in population size (Figure 2, Figure S4).

We shall focus rather on the distinction between the white regions of the parameter space, where no distinct clusters form and prevail over 200 generations, and the purple region, which indicates the tendency to form distinct clusters, rather than at the difference between, say, 5 and 20 clusters on average after 200 generations. The effects of variance constancy and magnitude are to some extent conflated. When both the constant contribution to variance (*η*) and the coefficient of proportional variability (*ν*) are small (consider, for example, a limiting case where *η* = 0 and *ν* = 0, or Supplementary Figure S14A) but homophily is very high, one can observe the emergence of distinct clusters. On the other hand, when we expect the offspring variance to be huge, even when it is due to very high *ν* and not *η*, clusters need not form if homophily is not high enough (see Figure S14B).

The proportion of points that are indicated not to be a part of any cluster by HDBSCAN (i.e. the proportion of noise) corroborates the conclusion about parameter values that lead to clustering. In the purple region of Figure 2, very few points are marked as noise (see supplementary Figure S4 for the visualisation of this measure). Pearson's correlation between the number of clusters and the proportion of noise was −0.56 at the level of individual simulation runs and −0.65 at the level of parameter combination averages. The proportion of noise emphasises the area in which clustering consistently happens but the number of clusters is small (in Figure 2, this area is still very light). A lower number of clusters means larger clusters for a given population size, so the adherence of points to their clusters is higher and the probability that a point is identified as noise between distinct clusters is low (Figure S4).

Regardless of the constant offspring standard deviation *η*, smaller populations face a reduction of effective trait-space dimensionality (Supplement S4, Figure S2), which conforms to a line of empirical and theoretical work on cultural diversity and covariation in small-scale societies (Smaldino, Lukaszewski, von Rueden, & Gurven, 2019). The effective dimensionality also decreases when relative offspring variability *ν* is high. In smaller populations, unidimensional polarisation or variability loss is more likely, which conforms to the results of previous research (Derex, Beugin, Godelle, & Raymond, 2013; Powell, Shennan, & Thomas, 2009). We thus arrive at an interesting interplay between agent positions and the effective trait space, where one depends on the other and influences it at the same time. Our simulations thus show that the formation of diverse cultural systems, where individuals differ along multiple dimensions, requires larger populations.

We obtain qualitatively similar results using the algorithm with normalisation (Figure S15) with the exception of the more likely occurrence of low number of clusters in a model with low *ν* and intermediate *h* despite high *η* (Figure S19). The results of simulations with additional multidimensional noise were also very similar to those of the basic model (Figure S20). The extra noise only served as a potential source of agents bridging neighbouring clusters, so the domain with intensive clustering is smaller and shifted towards higher homophily values *h*.

## 4. Discussion

Over time, various models were proposed in an attempt to explain the formation of human groups with low intra-group and high inter-group cultural variance (Axelrod, 1997; Creanza & Feldman, 2014; Turner & Smaldino, 2018). Most of these models rely on concepts of conformity or the notion of discrete ethnic markers (Ross et al., 2018). The model presented here works with probability density functions and continuous culture space. It differs from the previously suggested models in one important aspect: whereas particulate models require the existence of mutually exclusive traits that can be employed as ethnic markers prior to the accumulation of cultural differences between groups, our model demonstrates the possibility of the emergence of distinct clusters regardless of the presence or absence of such markers. If distinct cultural groups form spontaneously within a continuum of agents and are only later recognised, labelled and possibly conspicuously marked, our model should be given a priority over the older models.

It is interesting to compare the presented results with works that demonstrate how cultural clusters can emerge from anti-conformity (Smaldino & Epstein, 2015). The model based on Durkheim's theory of counteraction between integrating (conformity towards the societal average) and individualising (tendency to differentiate more if there are too many self-similar individuals) forces in society yields clusters of agents if the individualising tendencies are strong (Mäs, Flache, & Helbing, 2010). Despite the superficial similarity of the manifested clusters, the models have opposite expectations about agents in densely/sparsely populated domains of culture space. By definition, the Durkheimian model assumes that agents in the densest domains deviate most from the mean value of their neighbours, while in the PVDI model with homophily such agents deviate the least, since they can choose from lot of self-similar agents, and the standard deviation is proportional to the distance between interacting agents. Apparently, functions of variance can secure clustering regardless of the direction of the association between variances in subsequent generations. While the Durkheimian model leads to pulsation between one and several unstable clusters, PVDI seems to render stability or the irreversible merging of clusters. It is possible that each of the two processes occurs at a different level of human social interaction. In the few-to-one or one-to-one transmission, PVDI may dominate, because the differences between sources of cultural information provide a perspective for adequate cultural differentiation, but when a person attempts to perceive society as a whole to adapt her role in it, Durkheim's individualisation may come into play.

Future studies should aim to combine both models for a whole new level of understanding of human subculture formation. More complex, e.g. polynomial functions between the variance of cultural inputs and the variance of cultural outputs may be also worth exploring.

Assortative interaction and proportionality between traits of the ‘parents’ and the ‘offspring’ are sufficient conditions for the formation of subcultures (see purple regions in Figure 2). The inability of a typical GP system, which approximates the model of polygenic additive inheritance, to form distinct varieties in the presence of assortative pairing alone might be interpreted as providing support for ecological theories of sympatric divergence (Mallet et al., 2009; Mank, 2009; Nosil, 2008; Rabeling, Schultz, Pierce, & Bacci, 2014; Schluter, 2009; Tyers et al., 2015). In the absence of any cultural inheritance, natural and sexual selection must take place concurrently to generate separate ecotypes or species (Gavrilets, 2014; Kondrashov & Kondrashov, 1999; Mank, 2009).

On the other hand, if an inheritance system, such as culture, of an unfragmented species of sexually reproducing organisms follows the PVDI, we must conclude that species homogeneity is due to stabilising natural selection or other external force. For instance, if we added a Gaussian fitness function that would decrease the probability that an individual is selected as a role model proportionally to its distance from the coordinates origin (point 0,0,…,0), homophily might be trumped by the convergence of all agents towards this point. In a system, where homophily is strong and the variance of outputs is proportional to the variance of inputs, sympatric speciation, however, should not be viewed as an exception but rather as a norm.

The problem with sympatric speciation is in the evolution of reproductive barriers in the presence of a geneflow: cultural differences between spontaneously emerging homogenous groups can potentially limit the geneflow between them and facilitate speciation. If there is then a potential for divergent selection, distinct groups can subsequently settle into distinct ecological niches (Cameron, 2003; Riesch, Barrett-Lennard, Ellis, Ford, & Deecke, 2012). Such clusters are precursors of cultures, subcultures, guilds, alternative subsistence strategies, political factions, etc. (Olsson & Paik, 2016). While biological species are usually viewed as stable, separate and enduring entities, subcultures may be only temporal (Chandler, 2020). On the other hand, the clusters that emerge in our model are also relatively stable, which clearly calls into question the persisting stress on the species level in research of structured biota (Pavlinov, 2013).

We suggest that the process of culturally facilitated clustering in trait space enables organisms with the capacity for social transmission to establish and maintain distinct ecotypes even prior to the emergence of any genetic differences between the groups (Mcelreath et al., 2003). Moreover, the behavioural clustering and limited information flow between groups enable the increase of species' realised niche (Derex, Perreault, & Boyd, 2018; Lazer & Friedman, 2007). Species like *Homo sapiens*, *Orcinus orca* or *Corvux corax* should be therefore capable of exploiting natural resources in ways which are out of the reach of organisms that rely solely on genetic inheritance. Sympatric fission, that is, cases where cultural divergence precedes any spatial dislocation of individuals, may play a vital role in the distribution of cultural variants across time and space (Olsson & Paik, 2016; Riesch et al., 2012). Gradually, one may see an accumulation of genetic differences aiding further schismogenesis (Roux et al., 2016). Cultural assortment sparked by preference for self-similar individuals may be the key factor that limits gene flow between emerging subpopulations. The attested historical adaptive radiations of hominins (Foley, 2002; Wood, 1992), cetaceans (Filatova & Miller, 2015; Morin et al., 2010; Riesch et al., 2012) and songbirds (Huber et al., 2007; Nottebohm, 1972; Vaneechoutte, 1997) may be in part due to the vast cultural capacity of these lineages.

## Supplementary material

For supplementary material accompanying this paper visit https://doi.org/10.1017/ehs.2023.19. click here to view supplementary material

## Research transparency and reproducibility

All code and the complete set of generated simulation data are available at: https://doi.org/10.17605/osf.io/pvyhe

## References

[ref1] Allaire, J., & Ushey, K. (2020). reticulate: R Interface to Python. https://github.com/rstudio/reticulate

[ref2] Axelrod, R. (1997). The dissemination of culture: A model with local convergence and global polarization. Journal of Conflict Resolution, 41(2), 203–226.

[ref3] Cameron, D. W. (2003). Early hominin speciation at the Plio/Pleistocene transition. HOMO – Journal of Comparative Human Biology, 54(1), 1–28. 10.1078/0018-442X-00057 12968420

[ref4] Campello, R. J. G. B., Moulavi, D., & Sander, J. (2013). Density-based clustering based on hierarchical density estimates. In J. Pei, V. S. Tseng, L. Cao, G. Xu, & H. Motoda (Eds.), Advances in knowledge discovery and data mining, part II (pp. 160–172). Springer.

[ref5] Cavalli-Sforza, L. L., & Feldman, M. W. (1976). Evolution of continuous variation: Direct approach through joint distribution of genotypes and phenotypes. Proceedings of the National Academy of Sciences of the United States of America, 73(5), 1689–1692. 10.1073/pnas.73.5.1689 1064041 PMC430365

[ref6] Cavalli-Sforza, L. L., & Feldman, M. W. (1978). The evolution of continuous variation. III. Joint transmission of genotype, phenotype and environment. Genetics, 90(2), 391–425. 17248869 10.1093/genetics/90.2.391 PMC1213897

[ref7] Cavalli-Sforza, L. L., & Feldman, M. W. (1981). Cultural transmission and evolution: A quantitative approach. Princeton University Press. 7300842

[ref8] Chakraborty, R., & Nei, M. (1982). Genetic differentiation of quantitative characters between populations or species. Genetics Research, 59(2), 303–314. 10.1038/hdy.1987.114 3679879

[ref9] Chandler, N. (2020). Neo-tribes or subcultures?: The nature of subcultures in large complex organizations. In Z. Nedelko & M. Brzozowski (Eds.), Recent advances in the roles of cultural and personal values in organizational behavior (pp. 18–35). IGI Global. 10.4018/978-1-7998-1013-1.ch002

[ref10] Claidière, N., & Sperber, D. (2007). The role of attraction in cultural evolution. Journal of Cognition and Culture, 7(1), 89–111. 10.1163/156853707X171829

[ref11] Claridge, M. F., Dawah, H. A., & Wilson, M. R. (1997). Practical approaches to species concepts for living organisms. In M. R. Claridge, H. A. Dawah, & M. R. Wilson (Eds.), Species: The units of biodiversity (pp. 1–16). Chapman & Hall.

[ref12] Coyne, J. A., & Orr, H. A. (2004). Speciation. Sinauer Associates.

[ref13] Creanza, N., & Feldman, M. W. (2014). Complexity in models of cultural niche construction with selection and homophily. Proceedings of the National Academy of Sciences of the United States of America, 111(Suppl. 3), 10830–10837. 10.1073/pnas.1400824111 25024189 PMC4113930

[ref14] Darwin, C. (1859). On the origin of species. John Murray.

[ref15] Derex, M., Beugin, M.-P., Godelle, B., & Raymond, M. (2013). Experimental evidence for the influence of group size on cultural complexity. Nature, 503(7476), 389–391. 10.1038/nature12774 24226775

[ref16] Derex, M., Perreault, C., & Boyd, R. (2018). Divide and conquer: Intermediate levels of population fragmentation maximize cultural accumulation. Philosophical Transactions of the Royal Society B: Biological Sciences, 373(1743). 10.1098/rstb.2017.0062 PMC5812974 29440527

[ref17] Dieckmann, U., & Doebeli, M. (1999). On the origin of species by sympatric speciation. Nature, 400(6742), 354–357. 10.1038/22521 10432112

[ref18] Edla, D. R., & Jana, P. K. (2012). A prototype-based modified DBSCAN for gene clustering. Procedia Technology, 6, 485–492. 10.1016/j.protcy.2012.10.058

[ref19] Ereshefsky, M. (2010). Darwin's solution to the species problem. Synthese, 175(3), 405–425. 10.1007/s11229-009-9538-4

[ref20] Filatova, O. A., & Miller, P. J. O. (2015). An agent-based model of dialect evolution in killer whales. Journal of Theoretical Biology, 373, 82–91. 10.1016/j.jtbi.2015.03.020 25817037

[ref21] Fisher, R. A. (1918). The correlation between relatives on the supposition of mendelian inheritance. Transactions of the Royal Society of Edinburgh, (52), 399–433.

[ref22] Foley, R. (2002). Adaptive radiations and dispersals in hominin evolutionary ecology. Evolutionary Anthropology, 11(S1), 32–37. 10.1002/evan.10051

[ref23] Gavrilets, S. (2004). Fitness landscapes and the origin of species. Princeton University Press.

[ref24] Gavrilets, S. (2014). Models of speciation: Where are we now? Journal of Heredity, 105(S1), 743–755. 10.1093/jhered/esu045 25149251

[ref25] Henrich, J. (2004). Demography and cultural evolution: How adaptive cultural processes can produce maladaptive losses – the Tasmanian case. American Antiquity, 69(2), 197–214.

[ref26] Henrich, J., & Boyd, R. (2002). On modeling cognition and culture, why cultural evolution does not require replication of representations. Journal of Cognition and Culture, 2(2), 87–112. 10.1163/156853702320281836

[ref27] Huber, S. K., De León, L. F., Hendry, A. P., Bermingham, E., & Podos, J. (2007). Reproductive isolation of sympatric morphs in a population of Darwin's finches. Proceedings of the Royal Society B: Biological Sciences, 274(1619), 1709–1714. 10.1098/rspb.2007.0224 PMC2493575 17504742

[ref28] Hubert, N., Calcagno, V., Etienne, R. S., & Mouquet, N. (2015). Metacommunity speciation models and their implications for diversification theory. Ecology Letters, 18(8), 864–881. 10.1111/ele.12458 26036711

[ref29] Jiang, Y., Bolnick, D. I., & Kirkpatrick, M. (2013). Assortative mating in animals. The American Naturalist, 181(6), E125–38. 10.1086/670160 23669548

[ref30] Jokinen, H., Florin, A.-B., Merilä, J., Momigliano, P., Fraimout, A., & Norkko, A. (2017). Extraordinarily rapid speciation in a marine fish. Proceedings of the National Academy of Sciences, 114(23), 6074–6079. 10.1073/pnas.1615109114 PMC5468626 28533412

[ref31] Kondrashov, A. S., & Kondrashov, F. A. (1999). Interactions among quantitative traits in the course of sympatric speciation. Nature, 400(1989), 351–354. 10.1038/22514 10432111

[ref32] Lazer, D., & Friedman, A. (2007). The network structure of exploration and exploitation. Administrative Science Quarterly, 52, 667–694.

[ref33] Luo, S. (2017). Assortative mating and couple similarity: Patterns, mechanisms, and consequences. Social and Personality Psychology Compass, 11(8), 1–14. 10.1111/spc3.12337

[ref34] Mallet, J., Meyer, A., Nosil, P., & Feder, J. L. (2009). Space, sympatry and speciation. Journal of Evolutionary Biology, 22(11), 2332–2341. 10.1111/j.1420-9101.2009.01816.x 19732264

[ref35] Mank, J. E. (2009). Sexual selection and darwin's mystery of mysteries. Science, 326(5960), 1639–1640. 10.1126/science.1184680 20019275

[ref36] Mäs, M., Flache, A., & Helbing, D. (2010). Individualization as driving force of clustering phenomena in humans. PLoS Computational Biology, 6(10). 10.1371/journal.pcbi.1000959 PMC2958804 20975937

[ref37] Mayr, E. (1947). Ecological factors in speciation. Evolution, 1(4), 263–288.

[ref38] Mcelreath, R., Boyd, R., & Richerson, P. J. (2003). Shared norms and the evolution of ethnic markers. Current Anthropology, 44(1), 122–130. 10.1086/345689

[ref39] Mesoudi, A., & O'Brien, M. J. (2008). The cultural transmission of great basin projectile-point technology II: An agent based computer simulation. American Antiquity, 73(4), 627–644. 10.2307/25470521

[ref40] Morin, P. A., Archer, F. I., Foote, A. D., Vilstrup, J., Allen, E. E., Wade, P., … Harkins, T. (2010). Complete mitochondrial genome phylogeographic analysis of killer whales (*Orcinus orca*) indicates multiple species. Genome Research, (858), 908–916. 10.1101/gr.102954.109.908 20413674 PMC2892092

[ref41] Nosil, P. (2008). Speciation with gene flow could be common. Molecular Ecology, 17(9), 2006–2008. 10.1186/1742-9994-4-11.Schmitt 18410295

[ref42] Nottebohm, F. (1972). The origins of vocal learning. The American Naturalist, 106(947), 116–140.

[ref43] Olofsson, H., Frame, A. M., & Servedio, M. R. (2011). Can reinforcement occur with a learned trait? Evolution, 65(7), 1992–2003. 10.1111/j.1558-5646.2011.01286.x 21729054

[ref44] Olofsson, H., & Servedio, M. R. (2008). Sympatry affects the evolution of genetic versus cultural determination of song. Behavioral Ecology, 19(3), 596–604. 10.1093/beheco/arn002

[ref45] Olsson, O., & Paik, C. (2016). Long-run cultural divergence: Evidence from the Neolithic Revolution. Journal of Development Economics, 122, 197–213. 10.1016/j.jdeveco.2016.05.003

[ref46] Pavlinov, I. Y. (2013). The species problem, why again? In I. Y. Pavlinov (Ed.), The species problem – ongoing issues (Vol. 2, p. 64). IntechOpen. 10.5772/32009

[ref47] Pedregosa, F., Varoquaux, G., Gramfort, A., Michel, V., Thirion, B., Grisel, O., … Duchesnay, E. (2022). 2.3 Clustering. https://scikit-learn.org/stable/modules/clustering.html

[ref48] Powell, A., Shennan, S., & Thomas, M. G. (2009). Late Pleistocene demography and the appearance of modern human behavior. Science, 324(5932), 1298–1301. 10.1126/science.1170165 19498164

[ref49] Rabeling, C., Schultz, T. R., Pierce, N. E., & Bacci, M. (2014). A social parasite evolved reproductive isolation from its fungus-growing ant host in sympatry. Current Biology, 24(17), 2047–2052. 10.1016/j.cub.2014.07.048 25155509

[ref50] RCoreTeam. (2019). R: A language and environment for statistical computing. R Foundation for Statistical Computing. https://www.r-project.org/

[ref51] Riesch, R., Barrett-Lennard, L. G., Ellis, G. M., Ford, J. K. B., & Deecke, V. B. (2012). Cultural traditions and the evolution of reproductive isolation: Ecological speciation in killer whales? Biological Journal of the Linnean Society. 10.1111/j.1095-8312.2012.01872.x

[ref52] Rosenberg, N. A., Mahajan, S., Ramachandran, S., Zhao, C., Pritchard, J. K., & Feldman, M. W. (2005). Clines, clusters, and the effect of study design on the inference of human population structure. PLoS Genetics, 1(6), 0660–0671. 10.1371/journal.pgen.0010070 PMC1310579 16355252

[ref53] Ross, C. T., Borgerhoff Mulder, M., Oh, S. Y., Bowles, S., Beheim, B., Bunce, J., … Ziker, J. (2018). Greater wealth inequality, less polygyny: Rethinking the polygyny threshold model. Journal of the Royal Society Interface, 15(147). 10.1098/rsif.2018.0752 PMC6073648 30021924

[ref54] Roux, C., Fraïsse, C., Romiguier, J., Anciaux, Y., Galtier, N., & Bierne, N. (2016). Shedding light on the grey zone of speciation along a continuum of genomic divergence. PLoS Biology, 14(12), 1–22. 10.1371/journal.pbio.2000234 PMC5189939 28027292

[ref55] Schluter, D. (2009). Evidence for ecological speciation and its alternative. Science, 323(February), 737–742. 19197053 10.1126/science.1160006

[ref56] Smaldino, P. E., & Epstein, J. M. (2015). Social conformity despite individual preferences for distinctiveness. Royal Society Open Science, 2(3). 10.1098/rsos.140437 PMC4448825 26064615

[ref57] Smaldino, P. E., Lukaszewski, A., von Rueden, C., & Gurven, M. (2019). Niche diversity can explain cross-cultural differences in personality structure. Nature Human Behaviour, 3(12), 1276–1283. 10.1038/s41562-019-0730-3 31527682

[ref58] Smith, J. M. (1966). Sympatric speciation. The American Naturalist, 100(916), 637–650.

[ref59] Sperber, D. (1996). Explaining culture. Blackwell.

[ref60] Stanton, J. M. (2001). Galton, Pearson, and the peas: A brief history of linear regression for statistics instructors. Journal of Statistics Education, 9(3), 1–13. 10.1080/10691898.2001.11910537

[ref61] Tureček, P., Slavík, J., Kozák, M., & Havlíček, J. (2019). Non-particulate inheritance revisited: Evolution in systems with parental variability-dependent inheritance. Biological Journal of the Linnean Society, 127(2), 518–533.

[ref62] Turner, M. A., & Smaldino, P. E. (2018). Paths to polarization: How extreme views, miscommunication, and random chance drive opinion dynamics. Complexity, 2018, 1–17. 10.1155/2018/2740959

[ref63] Tyers, A. M., Malinsky, M., Ngatunga, B. P., Genner, M. J., Turner, G. F., Terai, Y., … Challis, R. J. (2015). Genomic islands of speciation separate cichlid ecomorphs in an East African crater lake. Science, 350(6267), 1493–1498. 10.1126/science.aac9927 26680190 PMC4700518

[ref64] Van Der Walt, S., Colbert, S. C., & Varoquaux, G. (2011). The NumPy array: A structure for efficient numerical computation. Computing in Science and Engineering, 13(2), 22–30. 10.1109/MCSE.2011.37

[ref65] Vaneechoutte, M. (1997). Bird song as a possible cultural mechanism for speciation. Journal of Memetics – Evolutionary Models of Information Transmission, 1, 1–10.

[ref66] Van Rossum, G., & Drake, F. L. (2009). Python 3 reference manual. CreateSpace.

[ref67] Verzijden, M. N., Lachlan, R. F., & Servedio, M. R. (2005). Female mate-choice behavior and sympatric speciation. Evolution, 59(10), 2097–2108. 10.1111/j.0014-3820.2005.tb00920.x 16405155

[ref68] Via, S. (2001). Sympatric speciation in animals: The ugly duckling grows up. Trends in Ecology & Evolution, 16(7), 381–390. 11403871 10.1016/s0169-5347(01)02188-7

[ref69] Wood, B. (1992). Early hominid species and speciation. Journal of Human Evolution, 22(4–5), 351–365. 10.1016/0047-2484(92)90065-H

