# Supplementary material for: How subcultures emerge
Source: Evol Hum Sci. 2023 Jul 12;5:e24. doi: 10.1017/ehs.2023.19 (PMC10426082; doi:10.1017/ehs.2023.19)
Supplement: Supplementary file 1 [file S2513843X23000191sup.zip › S2513843X23000191sup001.docx]

Supplementary material

Contents

[Supplement S1. Derivation of Eq. (3) from Egs. (1) and (2) 2](#_Toc128323509)

[Supplement S2: Derivation of the formula that offspring point ends up between parental points in a system with pure PVDI 3](#_Toc128323510)

[Supplement S3. HDBSCAN algorithm description 4](#_Toc128323511)

[Supplement S4. Expected measurement values across the parameter space 6](#_Toc128323512)

[Supplement S5. Example simulation runs 9](#_Toc128323513)

[Supplement S6. Results of a model where agent distance is normalised after each step 18](#_Toc128323514)

[Supplement S7. Results with a random variation along all dimensions of the trait space 24](#_Toc128323515)

All Supplementary animations are deposited in a separate folder at

<https://doi.org/10.17605/osf.io/pvyhe>

# Supplement S1. Derivation of Eq. (3) from Egs. (1) and (2)

We have introduced the Galton-Pearson inheritance model in Eq. (1) and the PVDI model in Eq. (2) in the main article. We can easily combine the two by considering both mutation terms from (1) and (2) together

|  | $t_{o}=\mu\left( t_{p_{1}}, t_{p_{2}} \right)+N\left( 0,\eta^{2} \right)+N\left( 0,\nu^{2}\sigma^{2}\left( t_{p_{1}}, t_{p_{2}} \right) \right),$ |  |
| --- | --- | --- |

due to the additivity of variance

|  | $t_{o}=\mu\left( t_{p_{1}}, t_{p_{2}} \right)+N\left( 0,\eta^{2}+\nu^{2}\sigma^{2}\left( t_{p_{1}}, t_{p_{2}} \right) \right).$ |  |
| --- | --- | --- |

To generalise the model to a $D$-dimensional trait space, we consider agents represented by vectors $A\epsilon\mathbb{R}^{D}$ and replace the modulus in the definition of the standard deviation $\sigma\left( t_{p1}, t_{p2} \right)$ by a $D$-dimensional norm

|  | $\sigma\left( A_{p_{1}}, A_{p_{2}} \right)=\frac{\left\Vert A_{p_{1}}- A_{p_{2}} \right\Vert}{2},$ |  |
| --- | --- | --- |

where $A_{p_{1}},A_{p_{2}}\epsilon\mathbb{R}^{D}$ are parental agents.

The magnitude of the phenotypic mutation is therefore given by

|  | $N\left( 0,\eta^{2}+\nu^{2}\frac{\left\Vert A_{p_{1}}- A_{p_{2}} \right\Vert^{2}}{4} \right).$ | (S1) |
| --- | --- | --- |

Because we assume the offspring to be on the line connecting its parent agents $A_{p_{1}}$and $A_{p_{2}}$, the magnitude in Eq. (S1) needs to be applied to a unit vector parallel to the connection line. Since normal distribution is symmetric, we may use for instance the unit vector

|  | $\frac{A_{p_{1}}- A_{p_{2}}}{\left\Vert A_{p_{1}}- A_{p_{2}} \right\Vert}.$ |  |
| --- | --- | --- |

To obtain Eq. (3), all that remains is to add the resulting vector

|  | $\frac{A_{p_{1}}- A_{p_{2}}}{\left\Vert A_{p_{1}}- A_{p_{2}} \right\Vert}N\left( 0,\eta^{2}+\nu^{2}\frac{\left\Vert A_{p_{1}}- A_{p_{2}} \right\Vert^{2}}{4} \right)$ |  |
| --- | --- | --- |

to the $D$-dimensional mean $\mu(A_{p_{1}}, A_{p_{2}})$. We obtain

|  | $A_{o}=\mu(A_{p_{1}}, A_{p_{2}})+\frac{A_{p_{1}}- A_{p_{2}}}{\left\Vert A_{p_{1}}- A_{p_{2}} \right\Vert}N\left( 0,\eta^{2}+\nu^{2}\frac{\left\Vert A_{p_{1}}- A_{p_{2}} \right\Vert^{2}}{4} \right),$ |  |
| --- | --- | --- |

Which is Eq. (3).

# Supplement S2: Derivation of the formula that offspring point ends up between parental points in a system with pure PVDI

Recall that the cumulative distribution function of a random variable $X$ with normal distribution $N\left( m,\zeta^{2} \right)$ with mean $m$ and standard deviation $\zeta$ is

$$\Phi\left( x \right)=P\left( X\leq x \right)=\frac{1}{2}\left[ 1+\mathrm{erf}\left( \frac{x-m}{\zeta\sqrt{2}} \right) \right],$$

where $\mathrm{erf}$ is the Gauss error function defined as

$$\mathrm{erf}\left( z \right)=\frac{2}{\pi}\int_{0}^{z} e^{-t^{2}}dt.$$

It is well-known that $\mathrm{erf}$ is an odd function, i.e. $\mathrm{erf}\left( z \right)=-\mathrm{erf}\left( -z \right)$.

We are interested in the probability that offspring position ends up between parental positions. Without loss of generality, we can translate and rotate the vector connecting the parental positions so that we may consider only one-dimensional culture space and assume that the mean of the parental positions is at $m=0$. Let us denote the parental distance by $2d$, i.e. the parents are located at $\pm d$, respectively. As described in the main text, the position of the offspring, denoted here by a random variable $X$, is normally distributed with standard deviation $\sigma= \nu d$. The probability $p_{\mathrm{between}}$ that the position of the offspring remains between $-d$ and $d$ can be computed by

$$p_{\mathrm{between}}=P\left( -d<X\leq d \right)=\Phi\left( d \right)-\Phi\left( -d \right)=\frac{1}{2}\mathrm{erf}\left( \frac{d}{\nu d\sqrt{2}} \right)-\frac{1}{2}\mathrm{erf}\left( \frac{-d}{\nu d\sqrt{2}} \right)=\mathrm{erf}\left( \frac{1}{\nu\sqrt{2}} \right).$$

In particular, we observe that the probability $p_{between}$ that the offspring remains in the line segment limited by the positions of the parents depends only on the parameter $\nu$. Considered as a function of $\nu$, the probability $p_{\mathrm{between}}=p_{\mathrm{between}}(\nu)$ is a decreasing function.

# Supplement S3. HDBSCAN algorithm description

We will demonstrate the HDBSCAN clustering algorithm on the data in Fig S1A. The data were chosen similarly as in How HDBSCAN works (<https://hdbscan.readthedocs.io/en/latest/how_hdbscan_works.html>) from the documentation of the implementation of HDBSCAN in sklearn (Pedregosa et al., 2011). For comparison, we also classified the data using the well-known K-means clusterer with the correct number of clusters. The result in Fig. S1B shows that the K-means algorithm does not perform perfectly on clusters with non-circle geometry.

For two data points $a$ and $b$, we denote the distance between $a$ and $b$ by $d(a, b)$. 

**1. (estimate the density)** Given a positive integer $k$ and a point $a$, define the core distance ${core}_{k}(a)$ by the distance from $a$ to its $k$-th closest neighbour. The core distance corresponds to the density of data around a point $a$ by $1/{core}_{k}(a)$. In the HDBSCAN implementation in sklearn, the parameter $k$ is known as $min\_samples$.

**2. (construct distance reflecting both density and closeness)** Given $k$ as above and two points $a$ and $b$, define the mutual reachability distance $d_{mreach,k}\left( a,b \right)=max\left( \mathrm{cor}e_{k}\left( a \right),core\left( b \right),d\left( a,b \right) \right)$. The mutual reachability distance measures if two points are close to each other or are in a sufficiently dense region. In effect, points in sparse regions are more distant under the mutual reachability distance $d_{mreach,k}$ than under the original distance $d$ while the distance between points in dense regions is unaffected.

**3. (construct minimum spanning tree)** The minimum spanning tree is a tree connecting all the data points with the minimum possible total edge weight, where the weight of the edge is given by the distance of the two points under the mutual reachability distance.  (Fig S1C) The minimum spanning tree can be constructed by Jarník-Prim's algorithm (Rosen, 2011).

**4. (construct cluster hierarchy)** First, convert the minimum spanning tree into a single linkage tree. (Fig S1D) The single linkage tree can be viewed as a hierarchical version of the minimum spanning tree with the hierarchy determined by the weights of the edges in the minimum spanning tree with largest weights being on the top of the tree. Then, condense the single linkage tree into a cluster hierarchy: starting at the top of the tree, remove edges and keep only clusters that are larger than $min\_cluster\_size$, a given positive integer. (Fig S1E)

**5. (choosing the clusters)** It remains to choose the clusters from the cluster hierarchy. Intuitively, when deciding if a base cluster or its two descendants should be selected, one compares the volume of the clusters in the cluster hierarchy determined by its persistence. If the volume of the base cluster in the cluster hierarchy is larger than the sum of the volumes of the two descendants, choose the base cluster, otherwise choose the two descendants (ellipses in Fig S1E, Fig S1F, the hue of the point corresponds to the likelihood of being a part of a cluster).


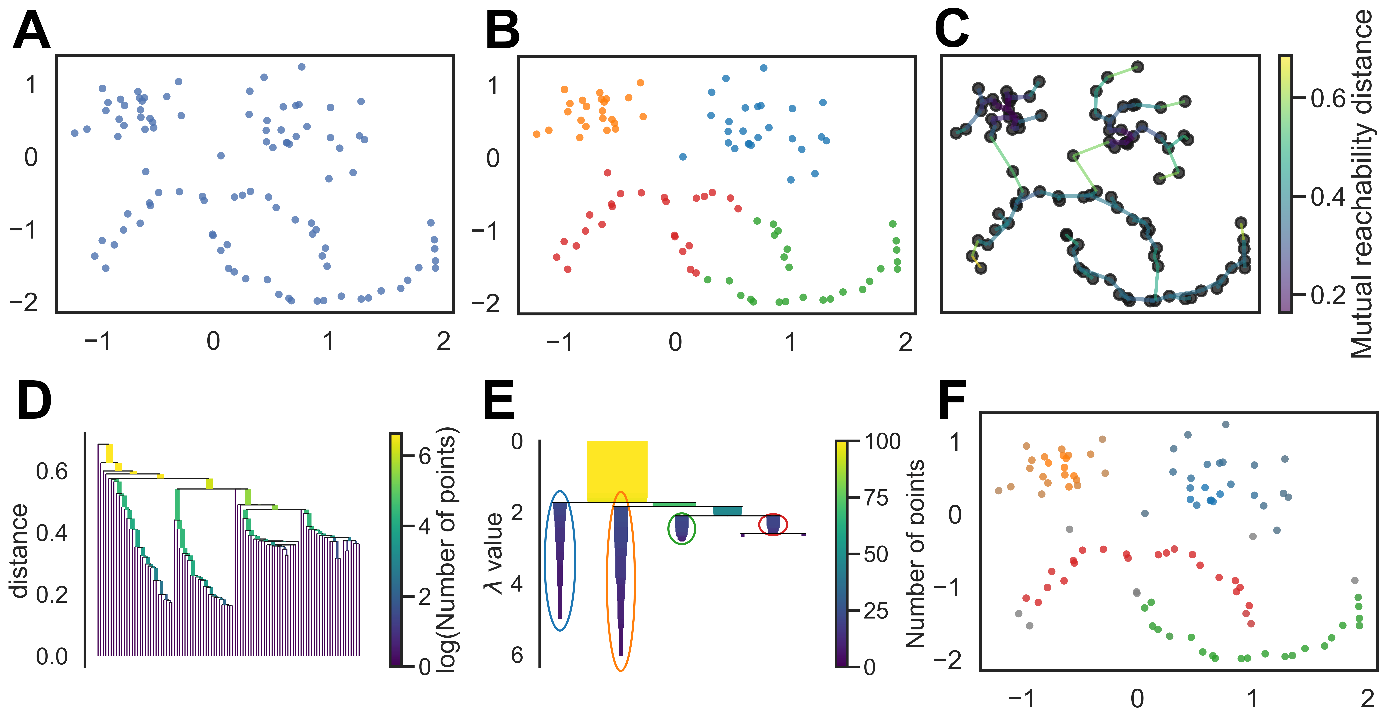


**Figure S1. Visulaization of the HDBSCAN algorithm,** A: exemplar data, B: result of K-means clustering, C: minimum spanning tree, D: cluster hierarchy, E: Selected stable clusters, F: the resulting point affiliation, see the description above for details.

**References:**

Pedregosa, F., Varoquaux, G., Gramfort, A., Michel, V., Thirion, B., Grisel, O., … Duchesnay, E. (2011). Scikit-learn: Machine learning in Python. *Journal of Machine Learning Research*, *12*, 2825–2830.

Rosen, K. (2011). *Discrete Mathematics and Its Applications* (7th ed.). New York: McGraw Hill.

# Supplement S4. Expected measurement values across the parameter space


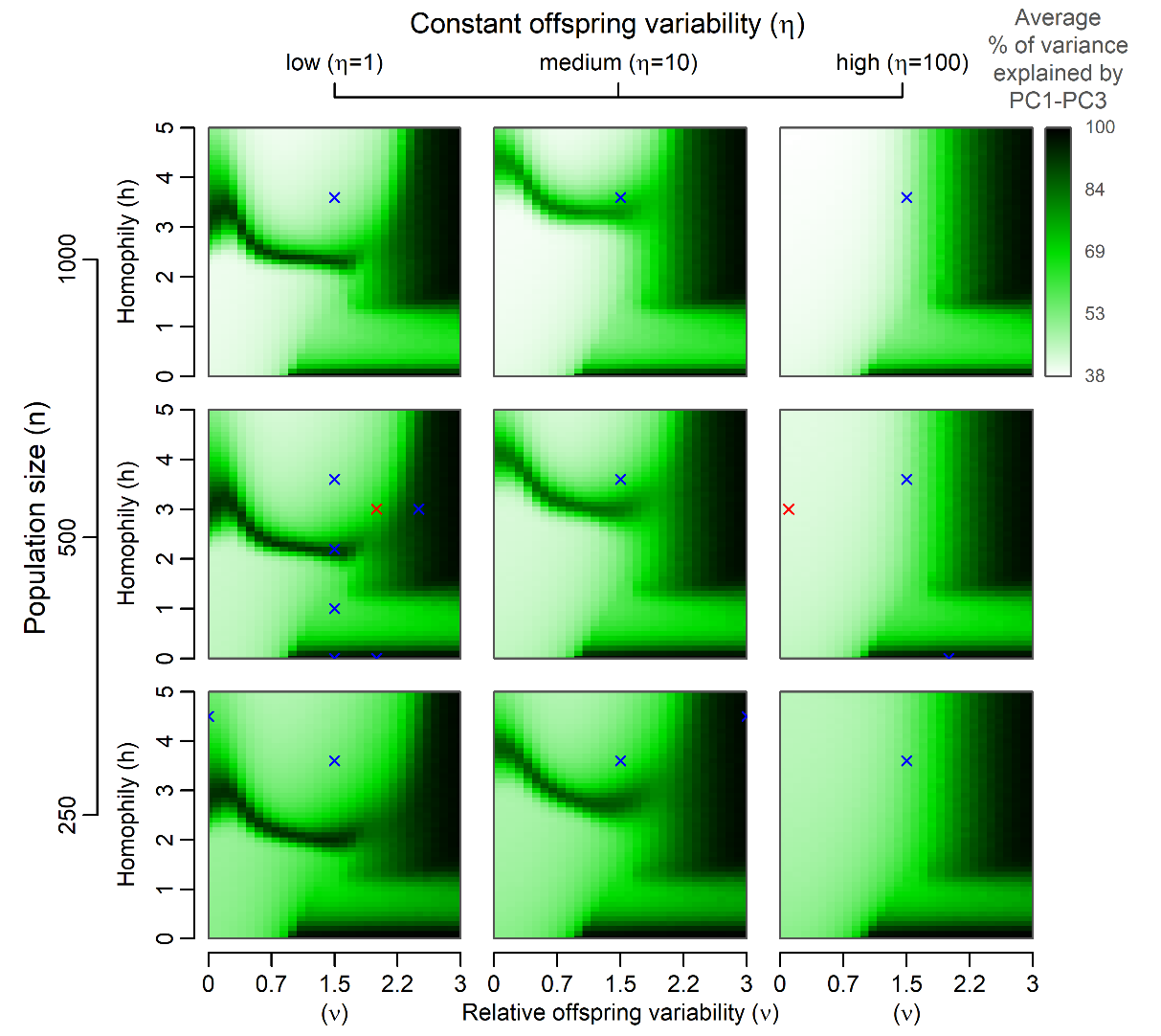


**Figure S2. A graphical summary of expected reduction in the number of effective dimensions** after 200 model generations. The same set of simulation runs as in Figure 2 was used to generate the image. PC1–PC3 stand for the first three principal components. Red crosses indicate the values of parameters used for single-run examples included in the main article. Blue crosses indicate examples available in the Supplementary material. The extreme values of explained variance (close to 100%) can stem from two distinct, yet not mutually exclusive, reasons. The “black bridge” on the left side of each panel corresponds to scenarios, where exactly two clusters are likely to emerge and exist for a substantial amount of the simulation run (see Figure S13). The dimension that separates the two clusters the best (PC1 after the rotation) obviously monopolizes almost all variance. The black region on the right of each panel is caused by an unconstrained variance growth ($\nu>2.10$ so two points are expected to get further apart if they are mutually selected as role models). Under such circumstances, variance along all effective dimensions of the point configuration is expected to grow exponentially, so the dimensions that are more prominent at the beginning by chance grow disproportionally faster in absolute terms and soon contain most of the extreme variance that emerges (Figure S7). Negative or low homophily can lead to a similar scenario even at lower values of $\nu$, because the variance is expected to grow for the population as a whole even if every two interacting points are expected to get closer to each other (Figure S9).


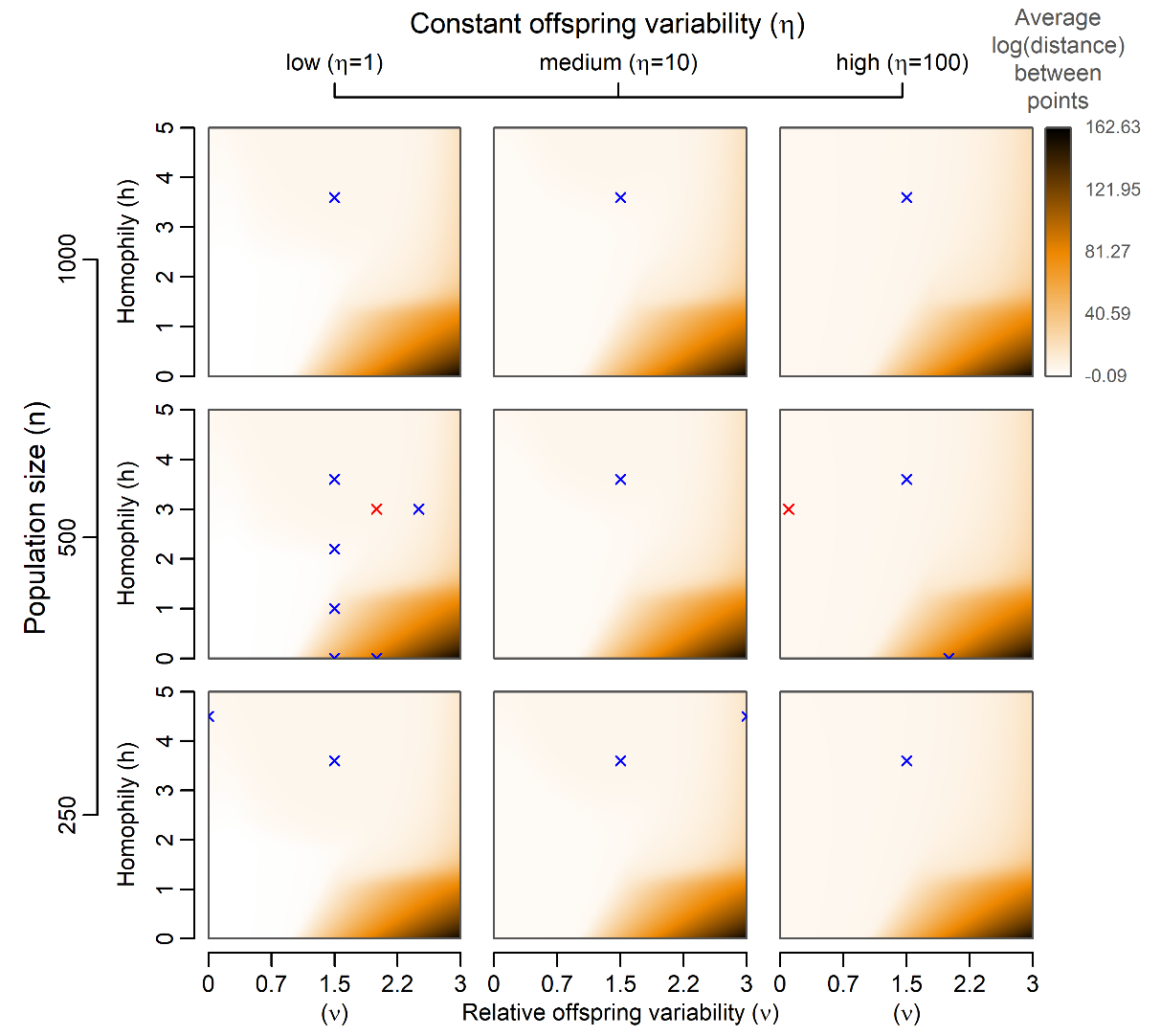


**Figure S3. A graphical summary of the expected average distance between agent positions in a culture-space** after 200 model generations. The same set of simulation runs as in Figure 2 was used to generate the image. Red crosses indicate the values of parameters used for single-run examples included in the main article. Blue crosses indicate examples available in the Supplementary materials. The extreme distance between points at ends of simulations in the bottom right corner of each panel of parameter space is due to the combination of three things: 1. There is no selection in the model that could trim extreme values. 2. High values of $\nu$ cause points to move further apart. 3. Low values of $h$ cause formation of more distance pairs of agents and, in turn, an increased speed of the variance growth.


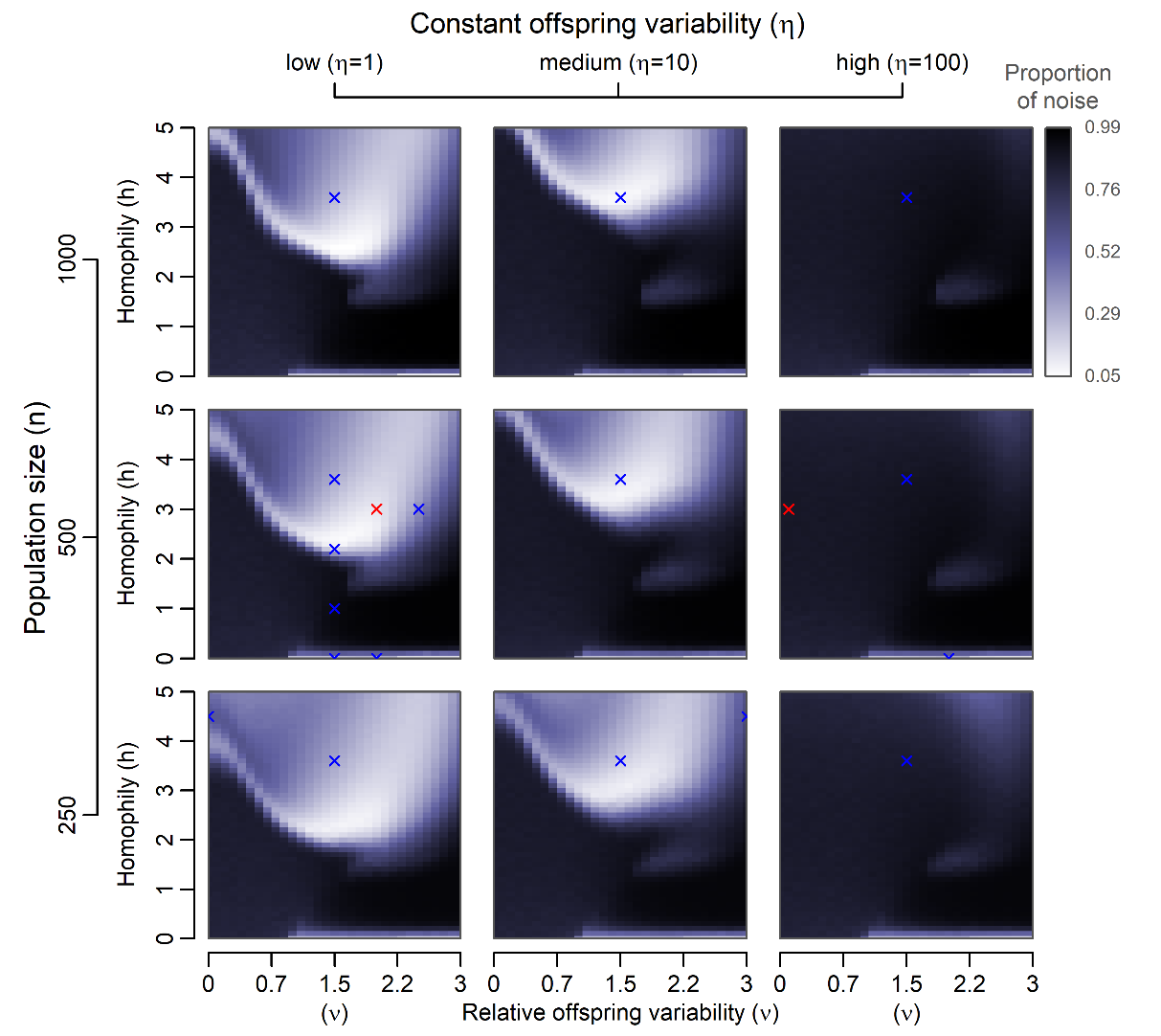


**Figure S4. A graphical summary of the proportion of noise (points that are not a part of any cluster)** after 200 model generations. The same set of simulation runs as in Figure 2 was used to generate the image. Red crosses indicate the values of parameters used for single-run examples included in the main article. Blue crosses indicate examples available in the Supplementary materials. The image is very similar to Figure 2. Where clustering conclusively happens, the noise proportion goes to 0. Where clustering is rare, noise proportion goes to 100%. The figure makes a region at low values of $\nu$ and high values of $h$ (it is darker here) stand out more than it does in Figure 1. This is the region where constant standard deviation $\eta$ matters more. Since noise generated due to $\eta$ is independent of the variance within clusters, more observations can end up between the tightly packed groups. This region is outlined by a lighter strip. The number of clusters in this part of parameter space is consistently low. Low number of clusters means higher total size of each cluster and hence a larger total variance. Larger clusters exhibit lower probability that their members that deviate more in absolute terms end up outside of the cluster and are identified as noise.

# Supplement S5. Example simulation runs


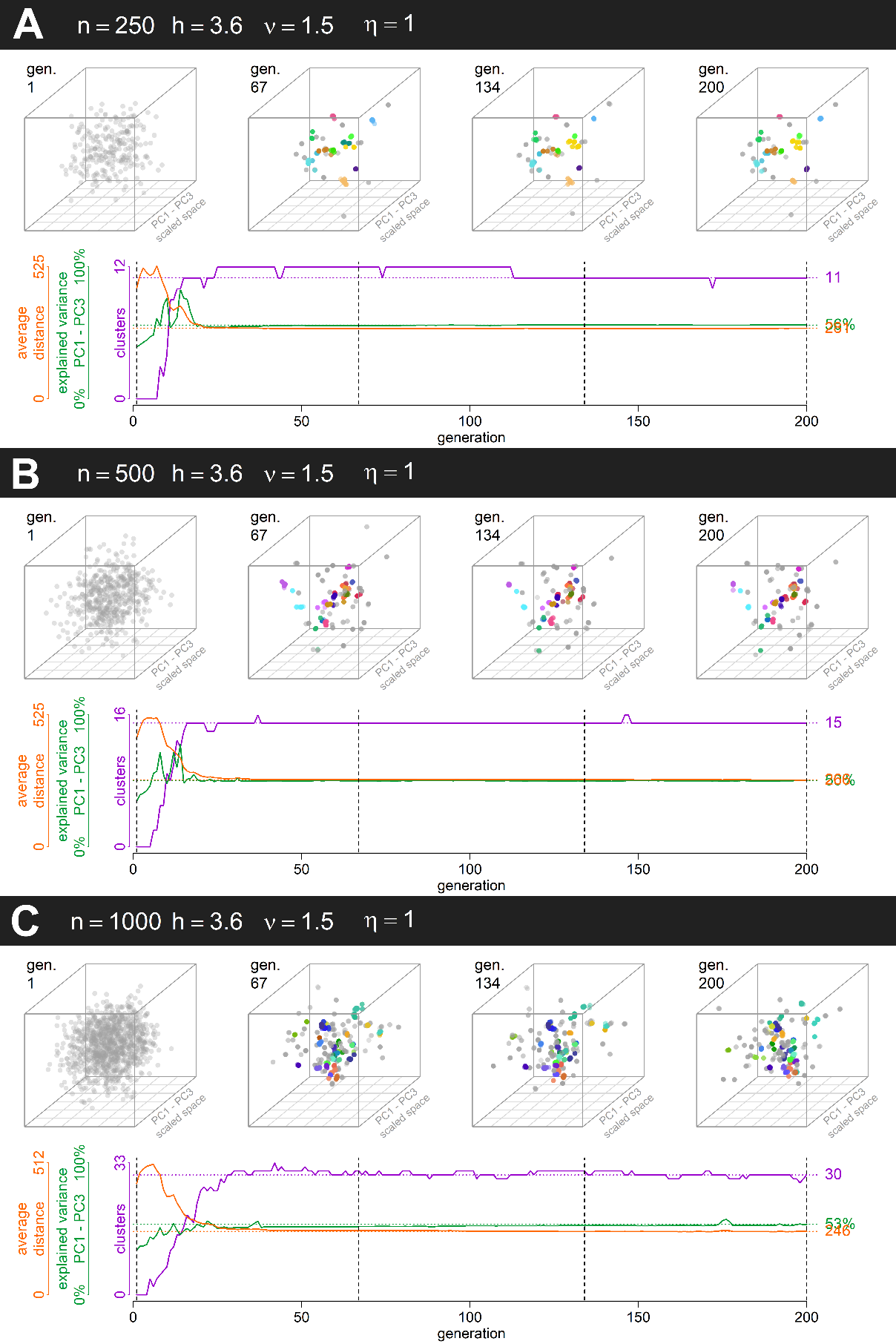


**Figure S5. Example simulations leading to stable distinct clusters, one simulation per small (A: n = 250), medium (B: n = 500), and large (C: n=1000) population.** All other parameters remain constant across the simulation runs (see Supplementary animations S05A–S05C).


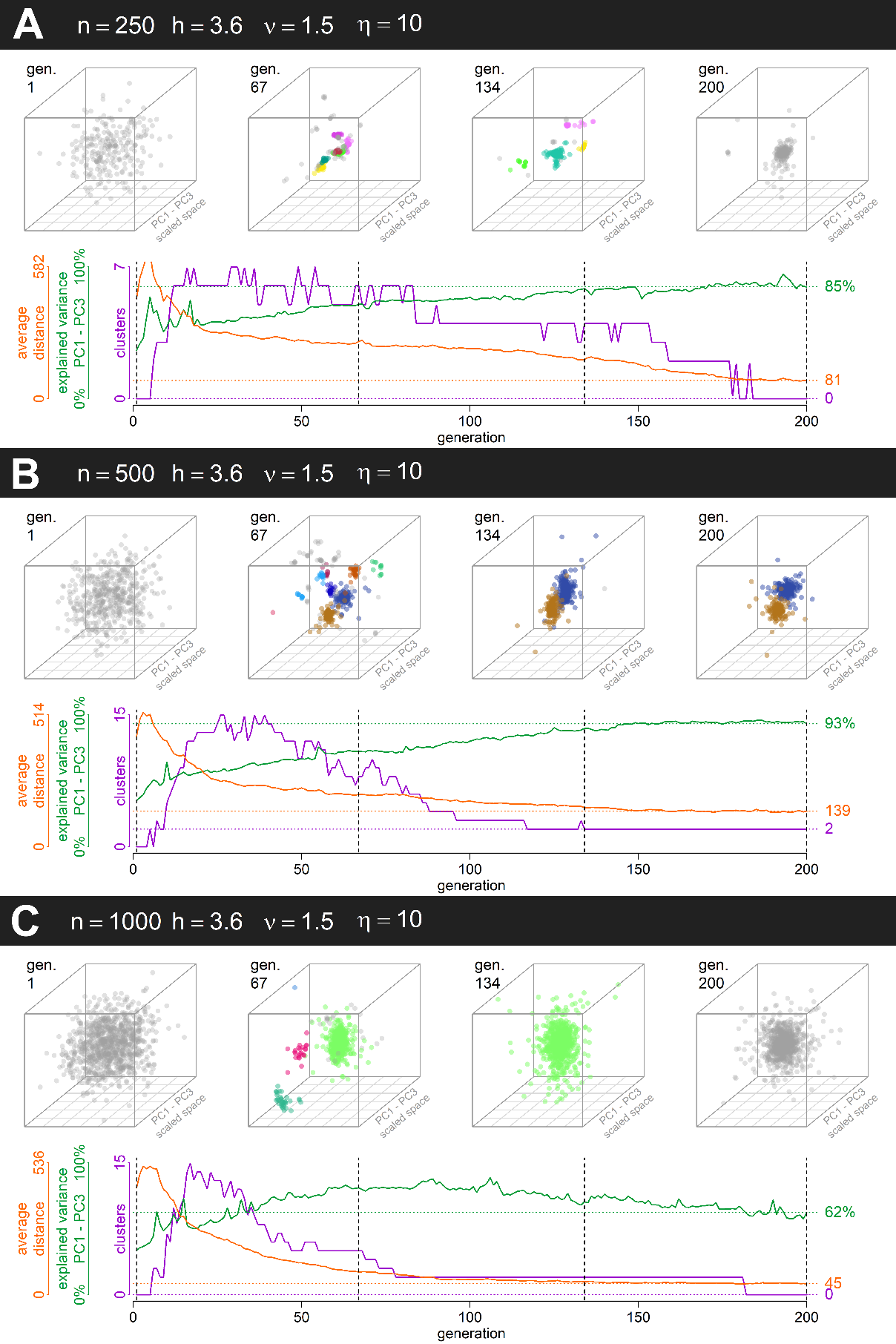


**Figure S6. Example simulations leading to stable distinct clusters, one simulation per small (A: n = 250), medium (B: n = 500), and large (C: n=1000) population** (see Supplementary animations S06A–S06C). We can clearly see that with a higher constant offspring standard deviation ($\eta$), clusters are less stable. They tend to merge and eventually disappear. (Here, we see regress to a single cluster in A and C, but a similar scenario is possible also for n = 500).


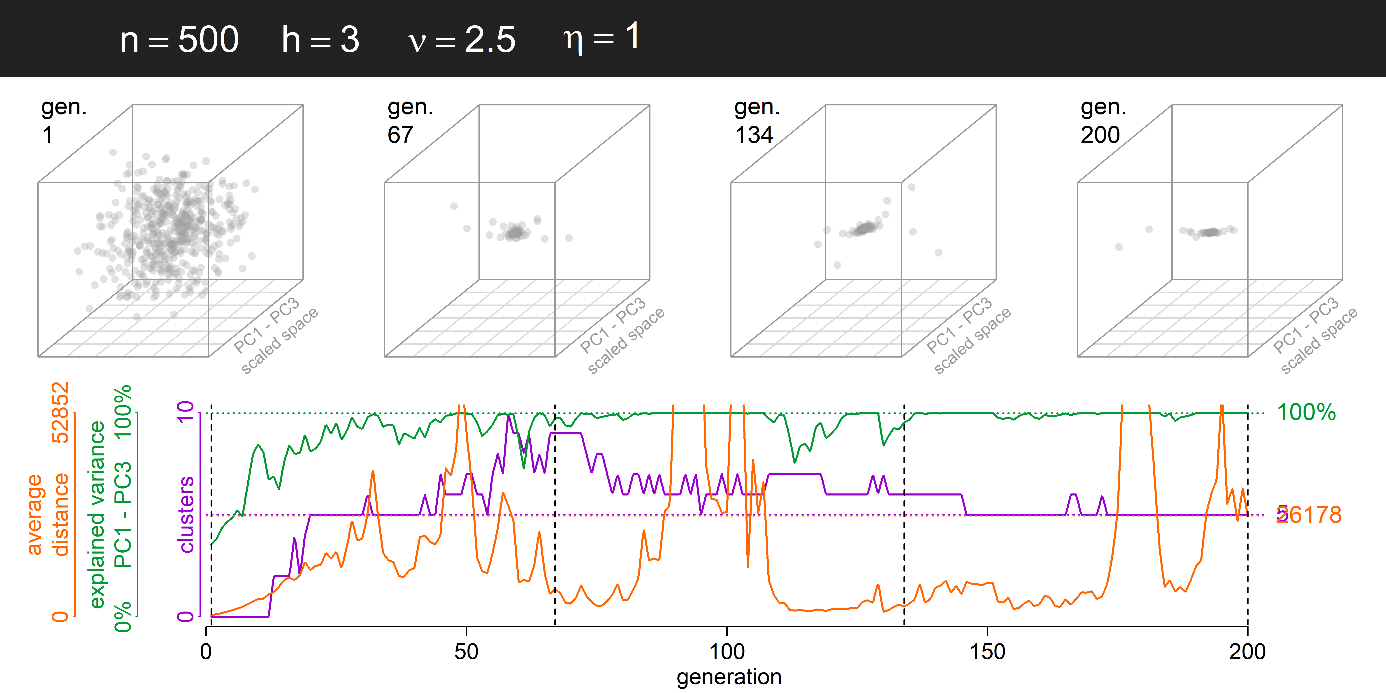


**Figure S7. Example simulation leading to a lower number of distinct clusters due to lower homophily (h = 3) and larger relative offspring variance (ν = 2.5)** (see supplementary animation S07).


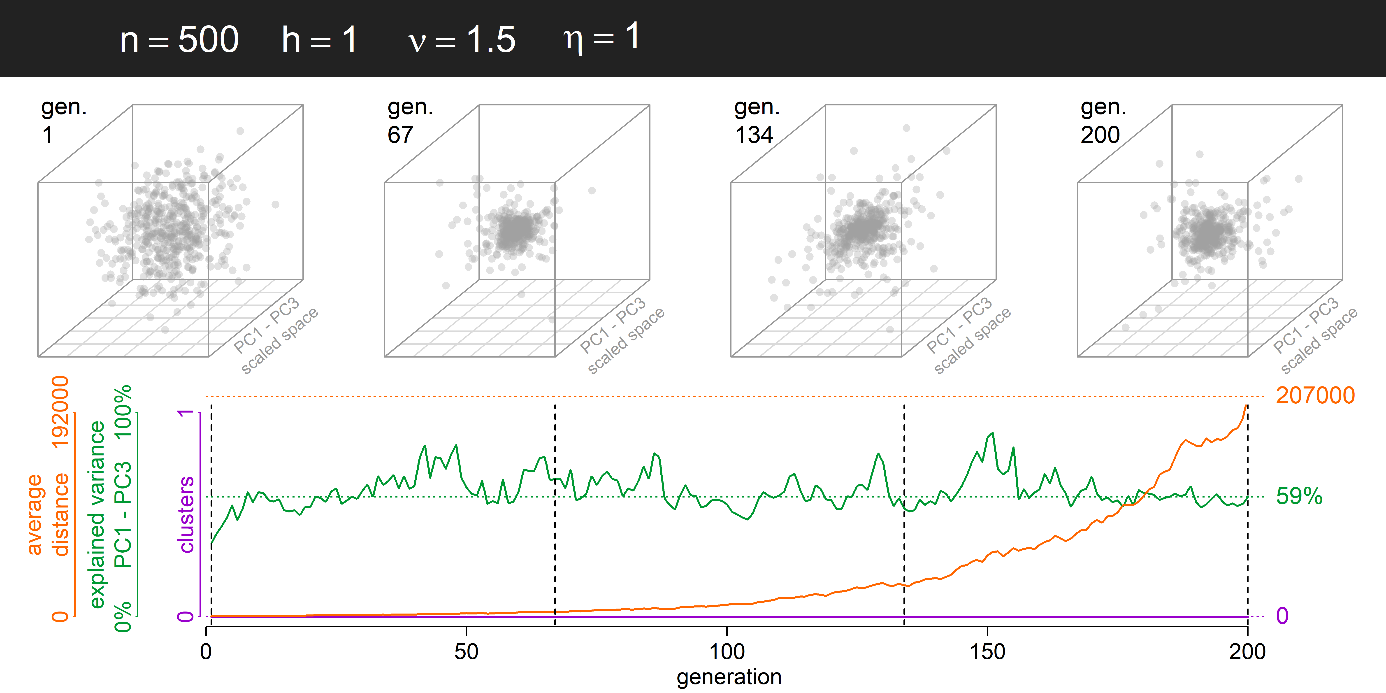


**Figure S8. Example simulation leading to a very low number of distinct clusters due to weak assortment between agents (h = 1)** (see supplementary animation S08).


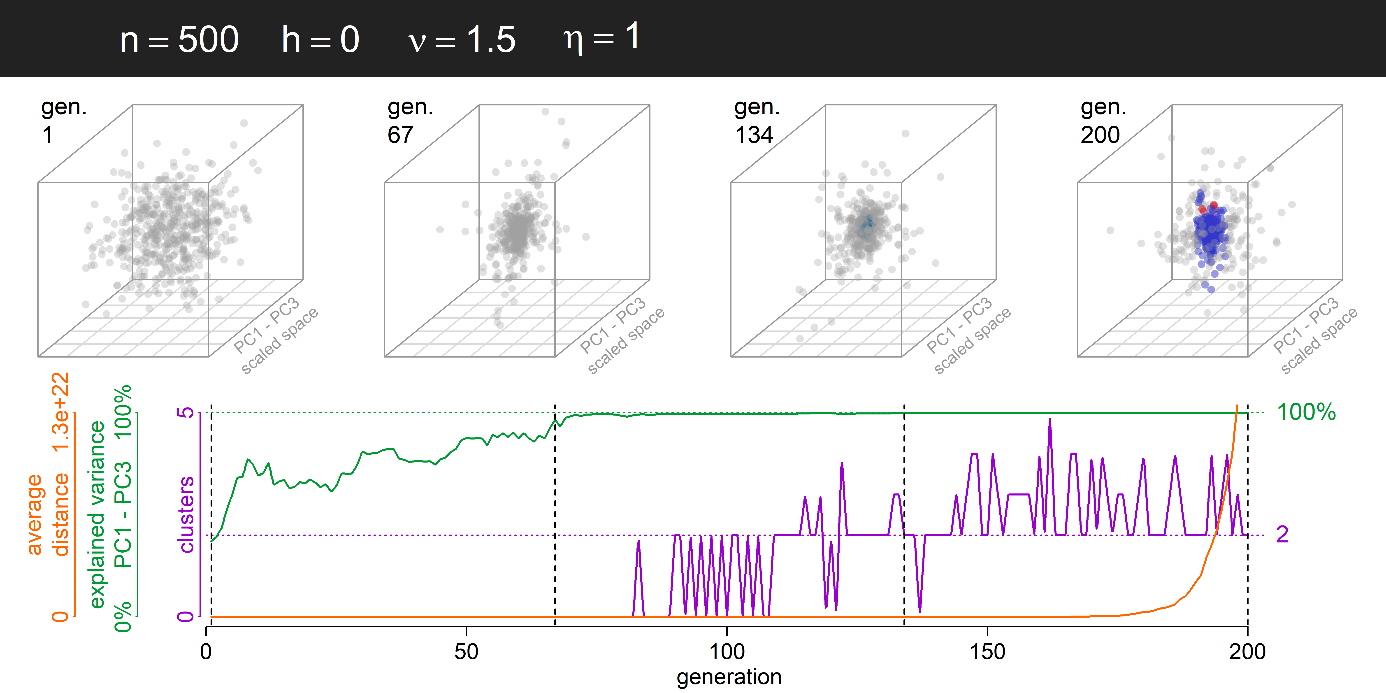


**Figure S9. Example simulation leading to variability explosion and weak clustering along a single dimension due to random interactions between agents (h = 0)** (see supplementary animation S09). Due to exponential variance explosion, it is in this case problematic to speak about the formation of subcultures, although the HDBSCAN algorithm is able to detect distinct clusters aligned along a single prominent culture-space dimension. In real life, of course, it is hard to imagine a trait, or a vector constituted by a combination of traits, along which variance (or average distance between points) could grow unrestricted to magnitude 10^22^. Results such as this should be interpreted in more general way: If homophily is low and coefficient for relative variance is high, we expect cultural variance to grow and the society to polarise. Notice, that the reduction of effective culture space dimensionality is present also in the modified version of the model, where the population configuration is normalised after every generation to hold the average distance between individuals constant (Figure S16).


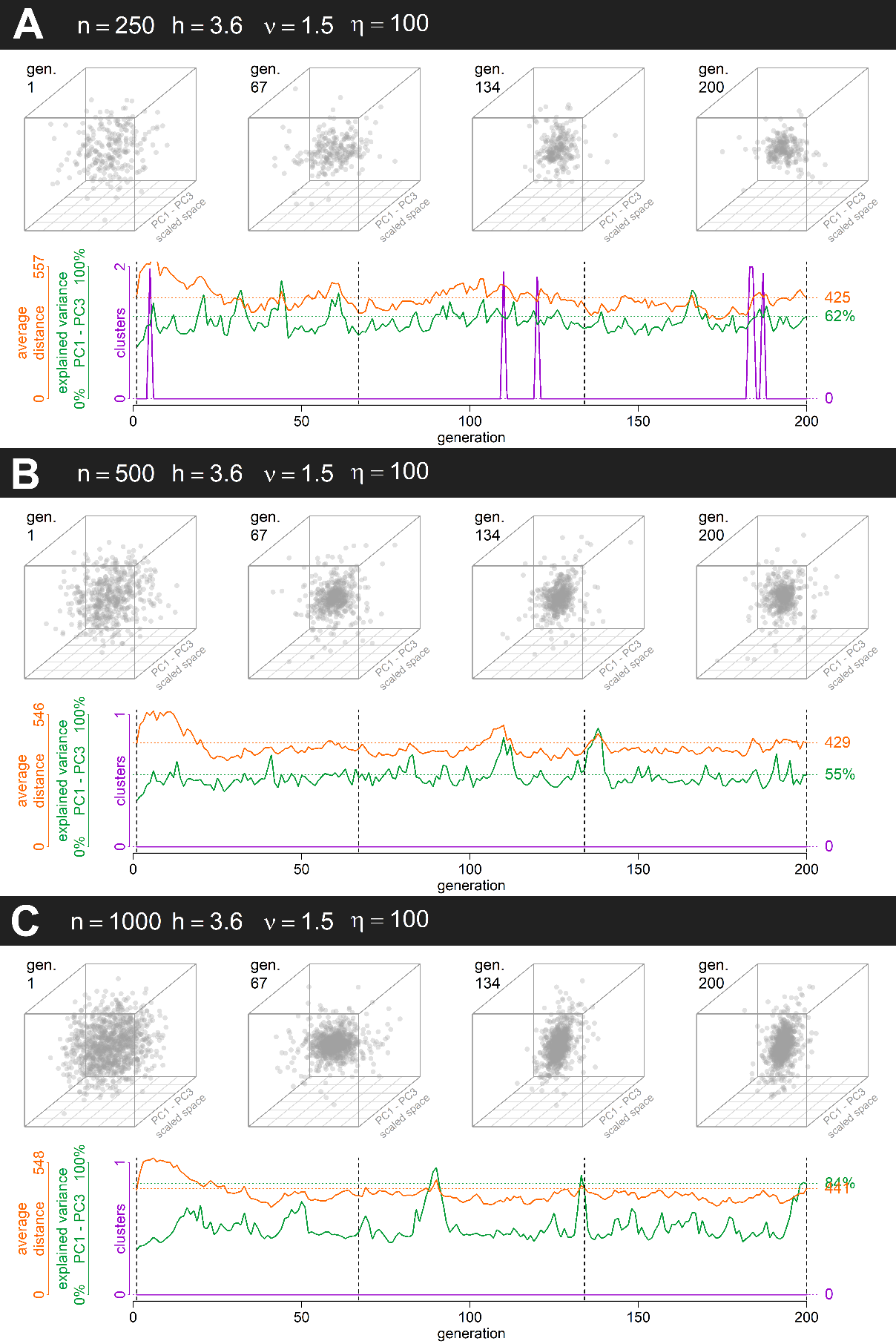


**Figure S10. Example simulations with a single stable cluster due to a dominant influence of Galton-Pearson inheritance (η = 100) despite high homophily (h = 3.6) and high relative offspring variance (ν = 1.5); one simulation per small (A: n = 250), medium (B: n = 500), and large (C: n=1,000) population** (see Supplementary animations S10A-S10C).


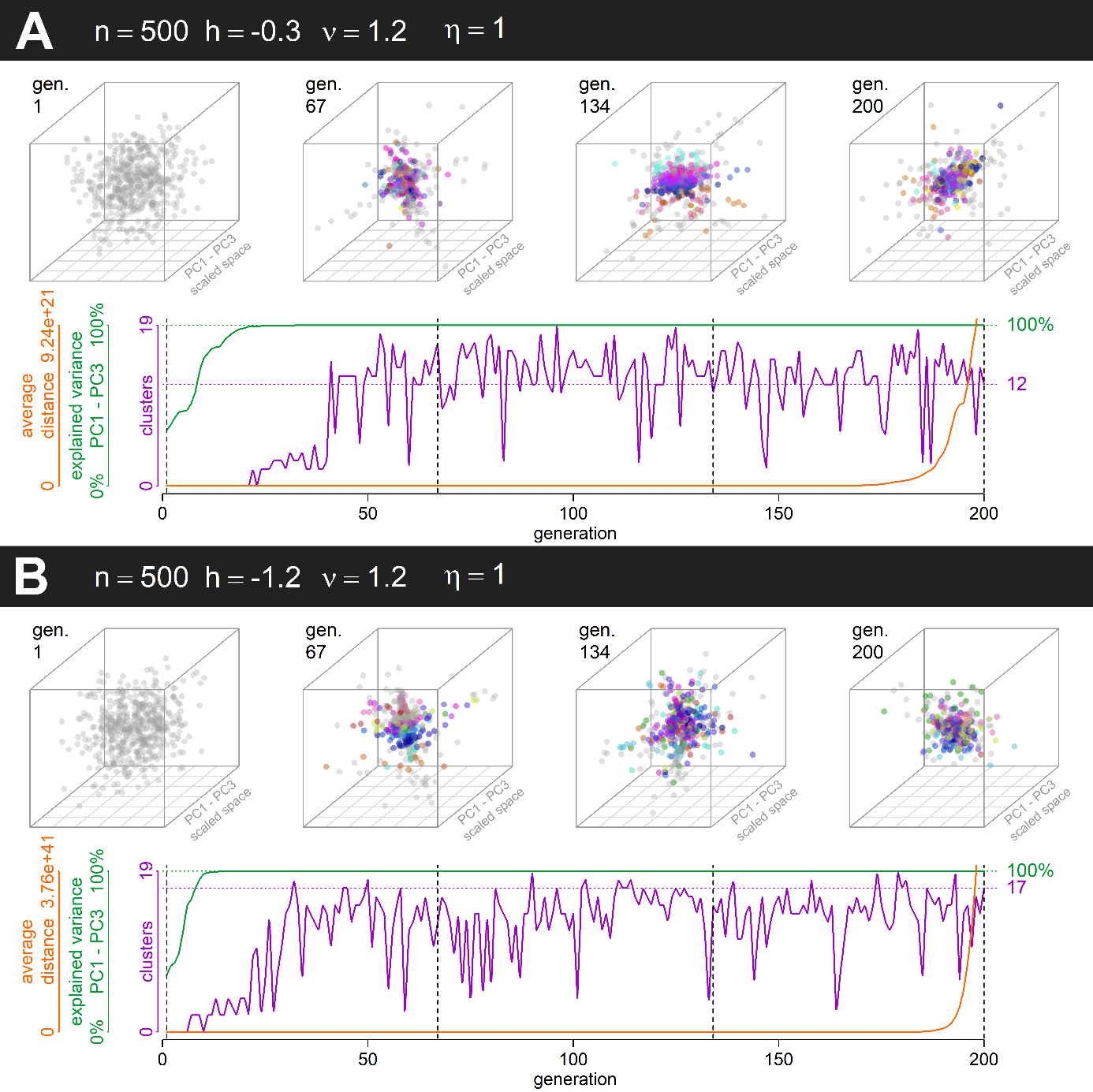


**Figure S11. Example simulations showing what happens when homophily is negative (A: h = -0.3, B: h = -1.2) and relative offspring variance is higher than 1 (ν = 1.2).** Due to the absence of stabilising selection (e.g., Gaussian fitness function decreasing the probability that an individual is selected as a role model proportionally to its distance from the coordinates origin), we see a rapid spread along a single effective dimension of the culture space (see Supplementary animations S11A and S11B). Due to the exponential variance explosion, it is in this case problematic to speak about the formation of subcultures, although HDBSCAN algorithm is able to detect distinct clusters aligned along a single prominent culture-space dimension.


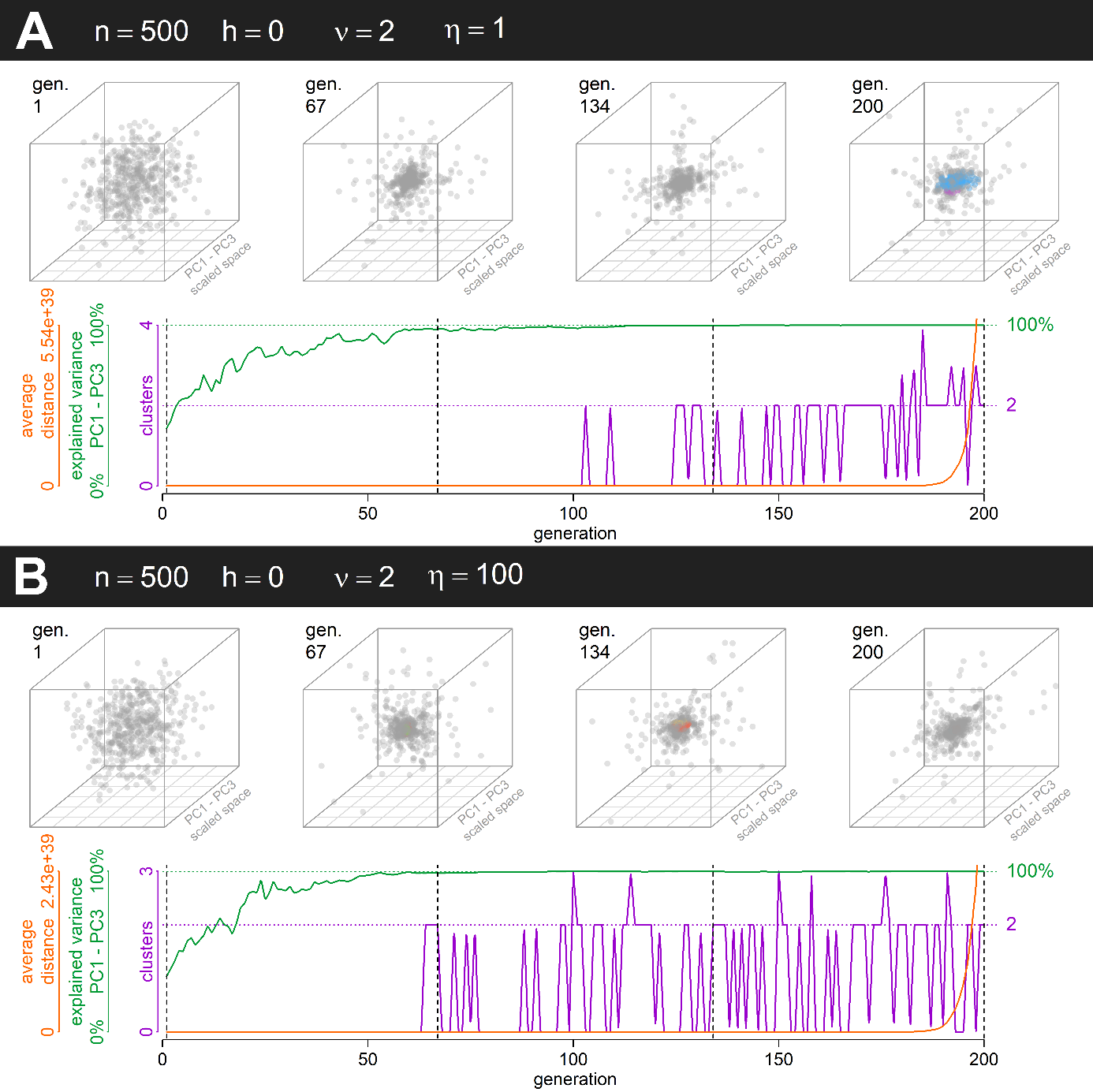


**Figure S12. Example simulations that show a rapid spread along a single effective culture space dimension despite random assortment.** This explosion is driven by a high relative offspring variance (ν = 2). In such case, the role of constant offspring variance (A: η = 1, B: η = 100) is negligible (see supplementary animation S12A and S12B). Due to exponential variance explosion, it is in this case problematic to speak about the formation of subcultures, although HDBSCAN algorithm is able to detect distinct clusters.


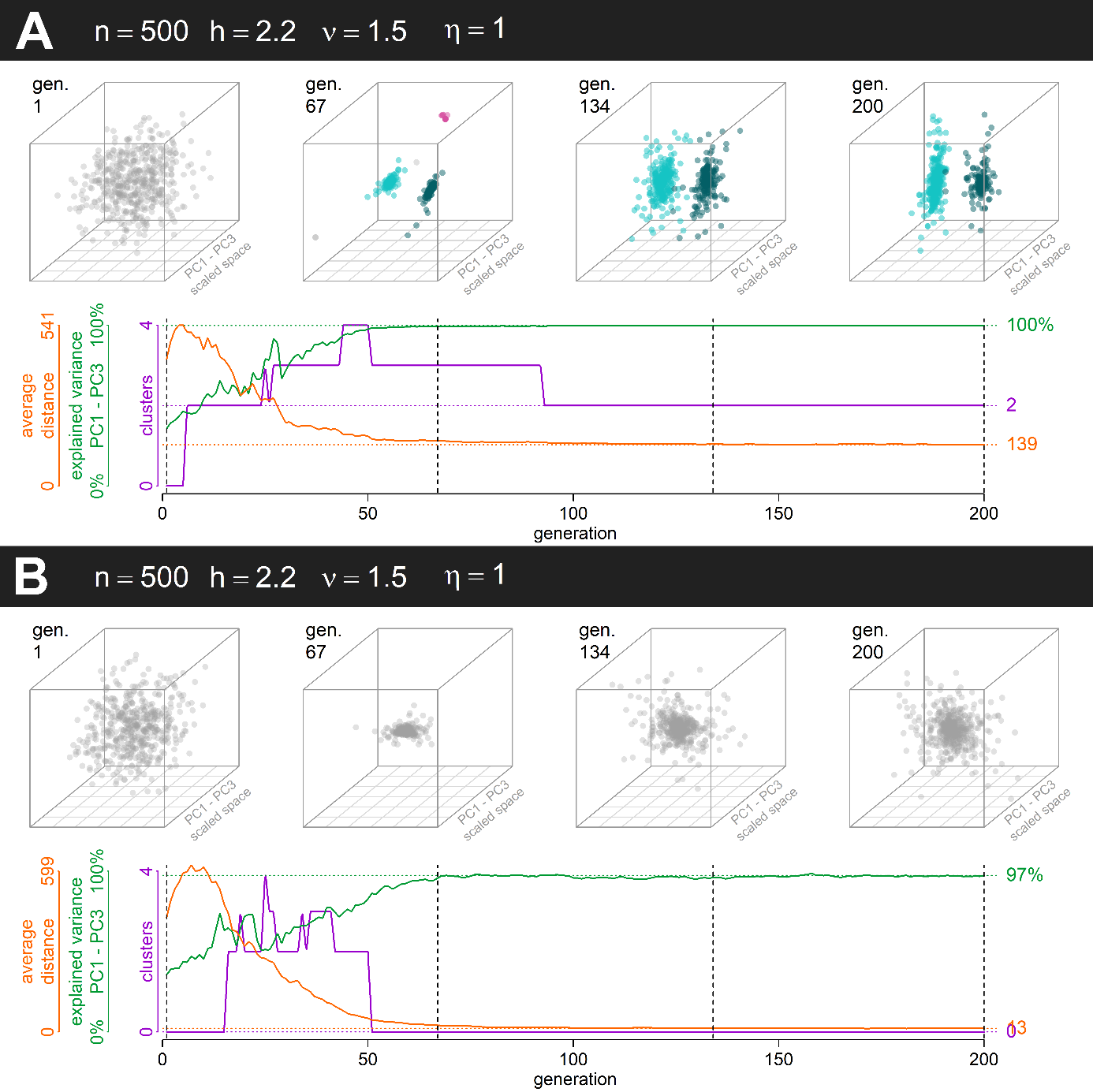


**Figure S13. Example simulation of a system with parameter combination that leads to a general loss of cultural variance but has a chance to form a small number of polarised clusters.** Such parameter combinations typically lie on the ‘black bridge’ apparent on panels for $\eta=1$ and $\eta=10$ in Figure S2. The reduction in dimensions is due to the emergence of just two stable clusters. This ‘polarisation of the population’ is typically reflected in a PC1 that monopolises most of the variance. In A, there emerged two distinct clusters which helped preserve some of the overall variance (see also the high proportion of variance explained by PC1–PC3). That was not, however, the universal outcome. All variance was lost where the population failed to form stable clusters (regardless of identical initial conditions). In such case, the population collapsed into a single point (see Supplementary animations S13A and S13B).


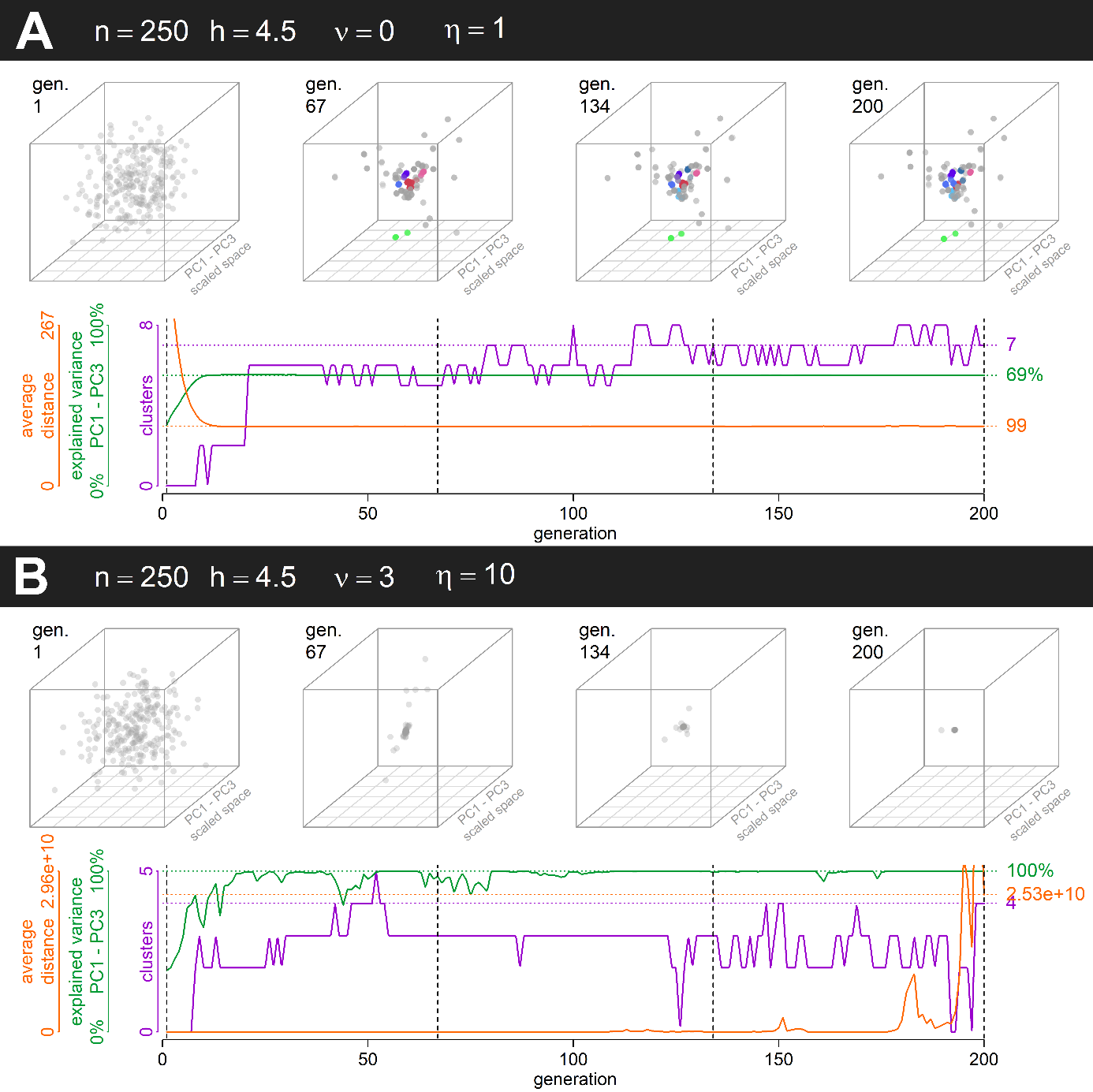


**Figure S14. Extreme simulation runs which demonstrate that a small** $\boldsymbol{\nu}$ **does not always prevent the emergence of clusters (A) when constant variance is small, and that excessively large** $\boldsymbol{\nu}$ **can in effect limit the number of clusters (B), because the overall new variance in each generation is huge despite** $\boldsymbol{\eta}$ **being reasonably small.** (See Supplementary animations S14A and S14B.)

# Supplement S6. Results of a model where agent distance is normalised after each step

The model presented in the main manuscript has the vast advantage of being simple and straightforward. It does not introduce any additional assumptions besides the pairing and inheritance algorithm. This simplicity, however, does have some side effects. For example, for many combinations of parameter values (when the configuration of agents’ position in the trait space is unconstrained), the basic system leads to one of two extreme configurations: in the first, all positions collapse into a tiny area within the trait space, where the pairing is as good as random (in case both $\eta$ and $\nu$ are small), or, in the second case, the configuration explodes to infinity along a single effective dimension of the trait space (in case the $\nu$ is excessively large or $\nu$ is positive and $h$ negative). Both of these extreme outcomes can be avoided if the entire configuration is normalised without distortion to maintain a constant average distance between agents in the trait space. This allows us to focus on changes in the relative configuration of agents without having to deal with variability loss or explosion. (In fact, though, a loss of all cultural variability may be a real possibility for the natural phenomena in question but, for the moment being, we can leave it aside.) Most findings, however, remain, unchanged regardless of the model.

In the results below, the methods are the same as in the main manuscript, only after each generation, the point coordinates are normalised to average distance of 436, which is approximately the average distance between normally distributed points in 10 dimensions with sd = 100 along each dimension.

**
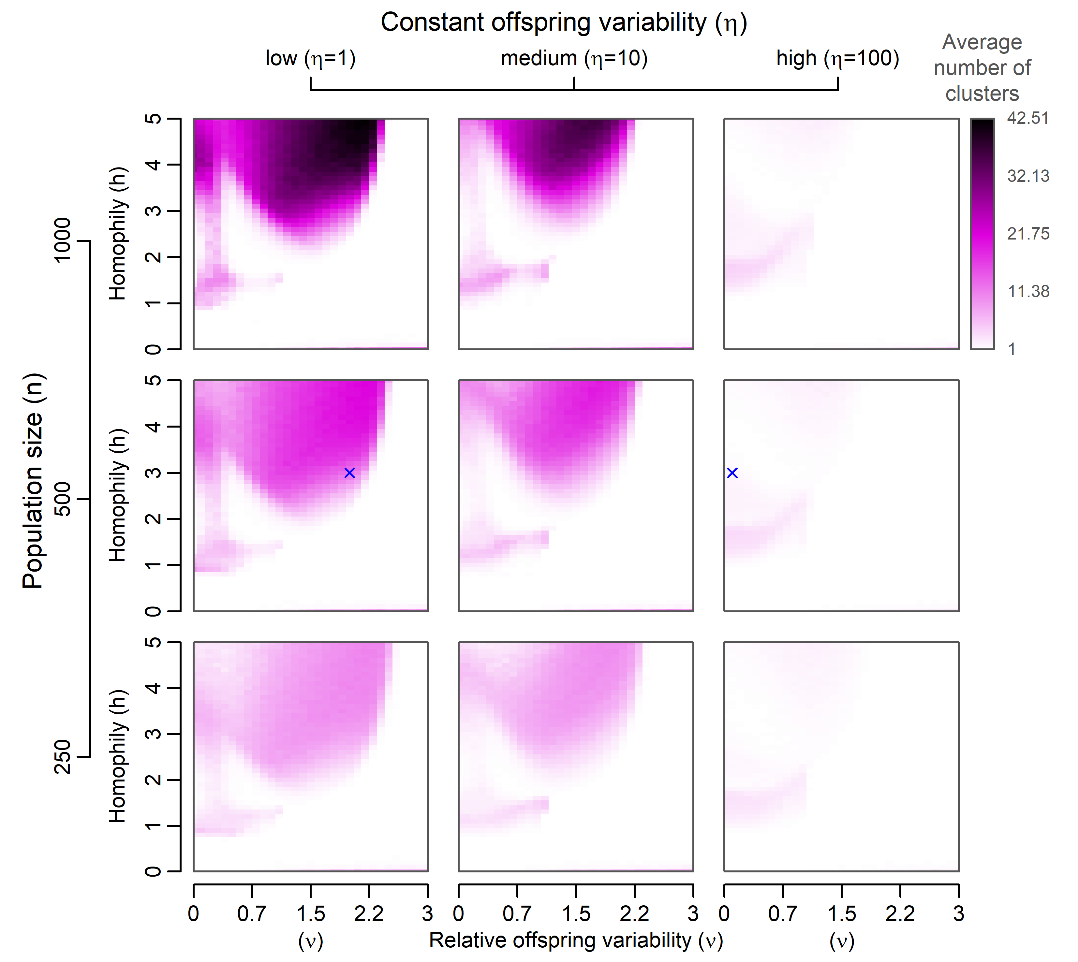
**

**Figure S15. A graphical summary of the tendency to form subcultures after 200 model generations in a system with normalised average distance**. The points in the 10-dimensional culture space were normally distributed across all dimensions at the beginning of each simulation run and 100 simulation runs were executed for each combination of parameters. Blue crosses indicate examples available below. Notice the new region in which clustering is possible: Region with intermediate homophily $h$ and low coefficient of proportional variability $\nu$. This is a region where polarisation along one dimension is expected (Figure S16) and there is a very little noise in between clusters (Figure S18), which means that this is an inflated “black bridge” from Figure S2, where emergence of two large clusters influences the outcome substantially. With normalisation occurring after each simulated generation, distances change proportionally to their magnitude. Similar to a system with PVDI and without normalisation, larger distances become larger, which may cause polarisation along a single dimension. Where intermediate homophily keeps points mostly within their clusters but scarce interactions across two clusters allow for the emergence of new variation along the dimension that connects them, the similarity between a system with PVDI and a system with low $\nu$ and normalisation is most prominent.


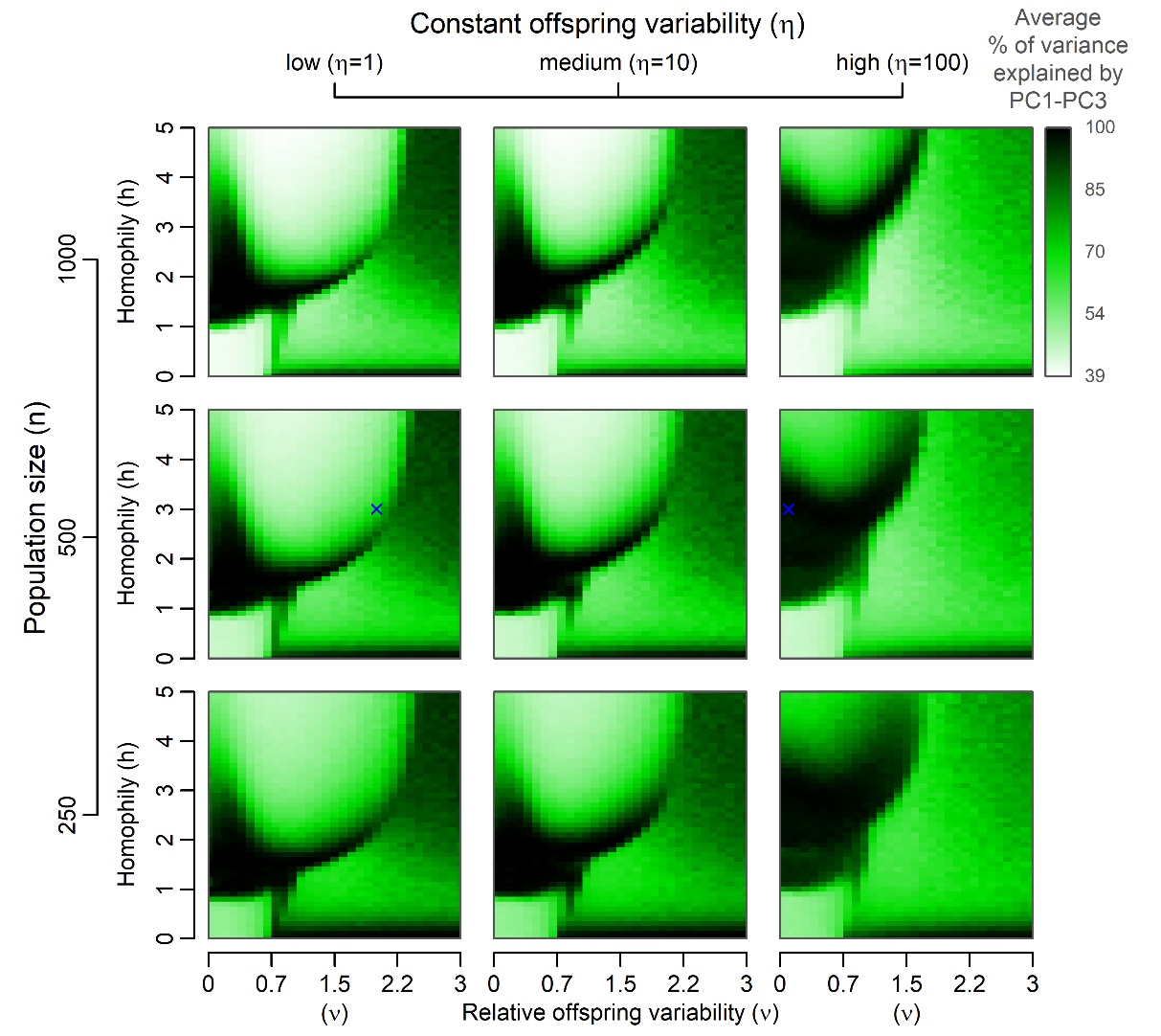


**Figure S16. A graphical summary of expected reduction in the number of effective dimensions after 200 model generations in a system with normalised average distance**. The same set of simulation runs as in Figure S15 was used to generate the image. PC1–PC3 stand for the first three principal components. See the explanation of dimension reduction in a system with large $\eta$ and small $\nu$ in the caption of Figure S19. Blue crosses indicate examples available below.


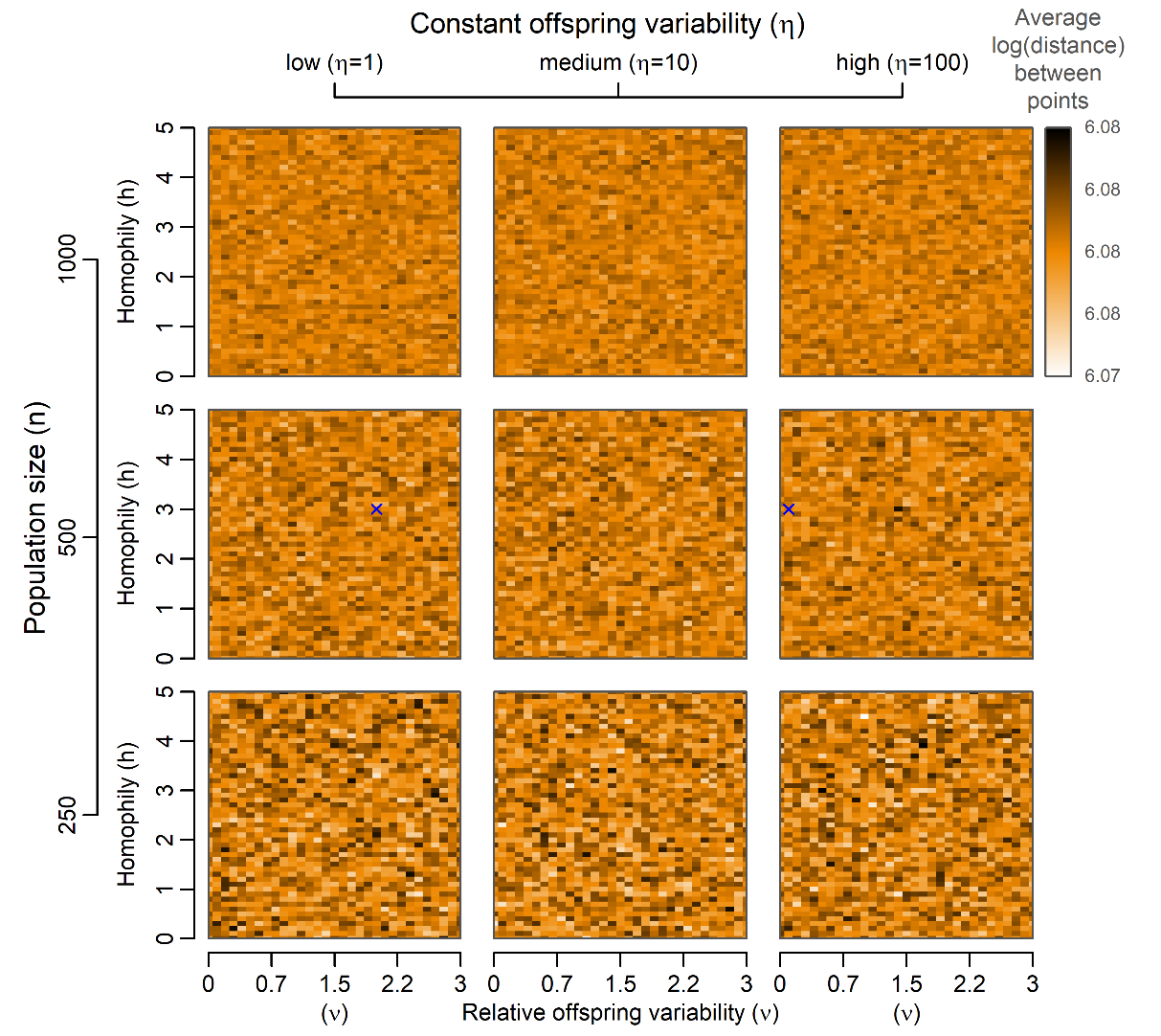


**Figure S17. A graphical summary of the expected average distance between agent positions in a culture space** **after 200 model generations in a system with normalised average distance**. The same set of simulation runs as in Figure S15 was used to generate the image. Blue crosses indicate examples available below.


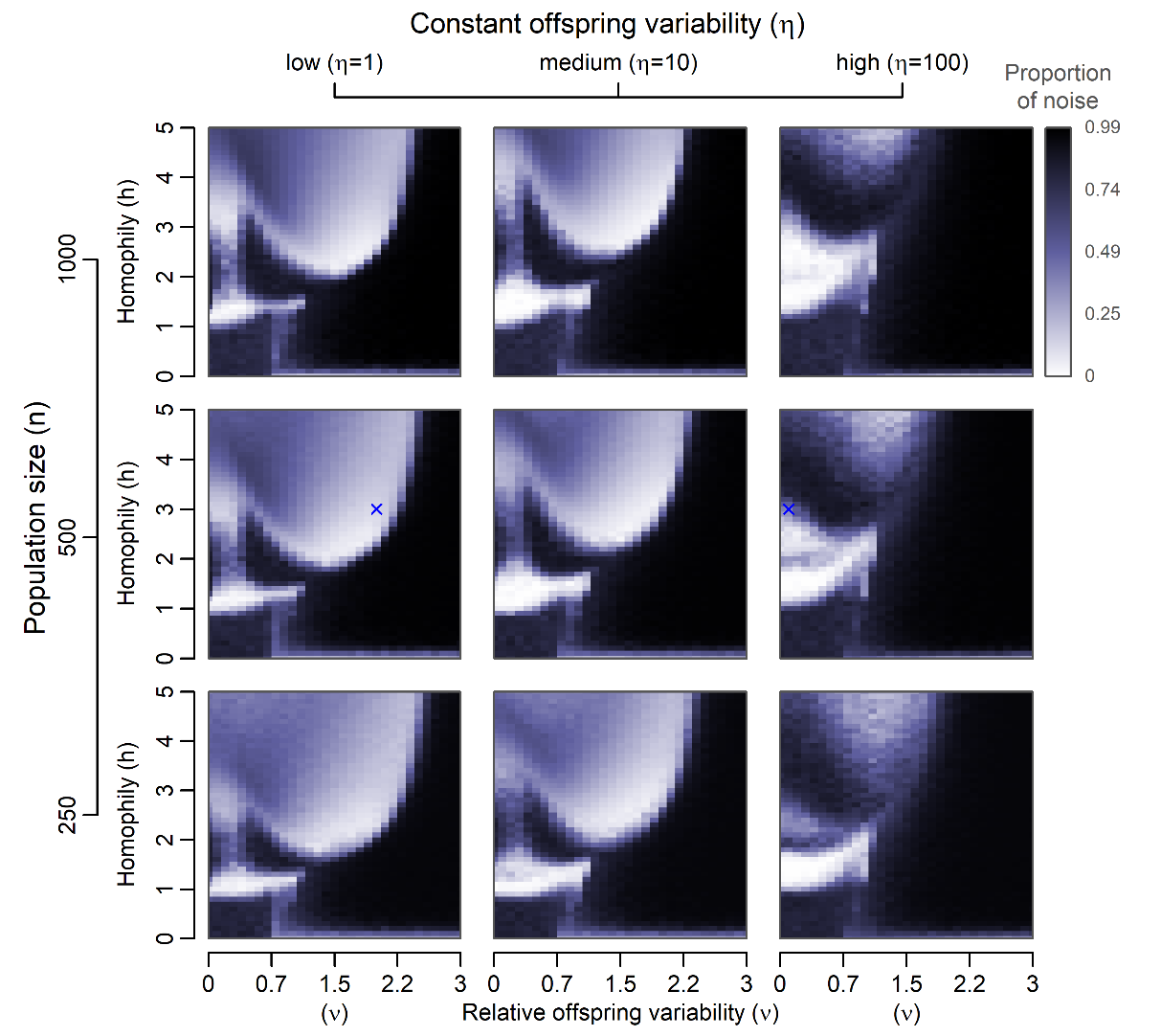


**Figure S18. A graphical summary of the proportion of noise (points that are not a part of any cluster)** after 200 model generations. The same set of simulation runs as in Figure 15 was used to generate the image. Blue crosses indicate examples available below.


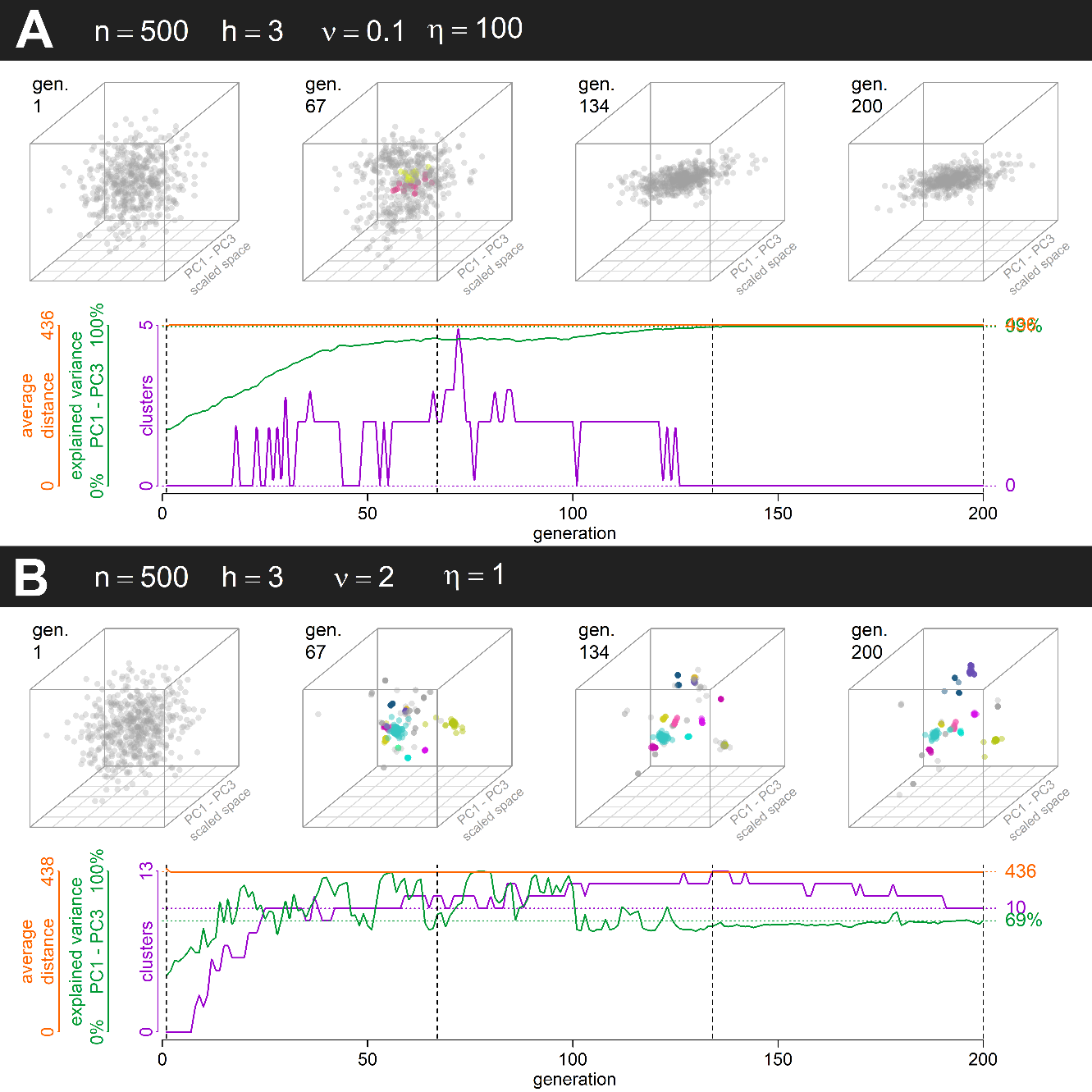


**Figure S19. Two simulation runs in a system with normalised average distance, one with a strong influence of Galton-Pearson inheritance (A) and one with a strong influence of PVDI (B).** For detailed description, see the caption of Figure 3 in the main article. (See Supplementary animations S19A and S19B.) Simulation run parameters were chosen to match those of Figure 3. For the sake of brevity, equivalents of Figures S5–S14 are not included but can be easily obtained using the code at <https://doi.org/10.17605/osf.io/pvyhe>

Notice the clustering tendencies and dimension reduction in a system with a large influence of Galton-Pearson inheritance and homophily. The polarisation is due to an inflation of the originally small differences that come up due to the normalisation after every generation (see also the explanation in the caption of Figure S15 and notice that this parameter combination appears on the edge of the new region of clustering which can be best seen in Figure S18).

# Supplement S7. Results with a random variation along all dimensions of the trait space

One could argue that a model where all random variation is realised along the vector linking parental positions is not realistic. It is trivial to modify the inheritance part of the model summarised in Eq. (3) of the main manuscript and add random variation along the D dimensions of the trait space, i.e.,

|  | $A_{o}=\mu\left( A_{p_{1}}, A_{p_{2}} \right)+\frac{A_{p_{1}}- A_{p_{2}}}{\left\Vert A_{p_{1}}- A_{p_{2}} \right\Vert}N\left( 0,\eta^{2}+\nu^{2}\frac{\left\Vert A_{p_{1}}- A_{p_{2}} \right\Vert^{2}}{4} \right)+X,$ |  |
| --- | --- | --- |

where $X=\left( {N\left( 0,\xi\right)}_{1},{N\left( 0,\xi\right)}_{2},\ldots,{N\left( 0,\xi\right)}_{D} \right) \epsilon\mathbb{R}^{D}$ with $\xi$ being a random phenotypic mutation of the offspring independent of parental positions.

The assumption of a larger variation (proportional to the parental distance for $\nu\neq0$) along the vector connecting parental positions is preserved.

Results after this slight modification for $\xi=1$ are included below.

**
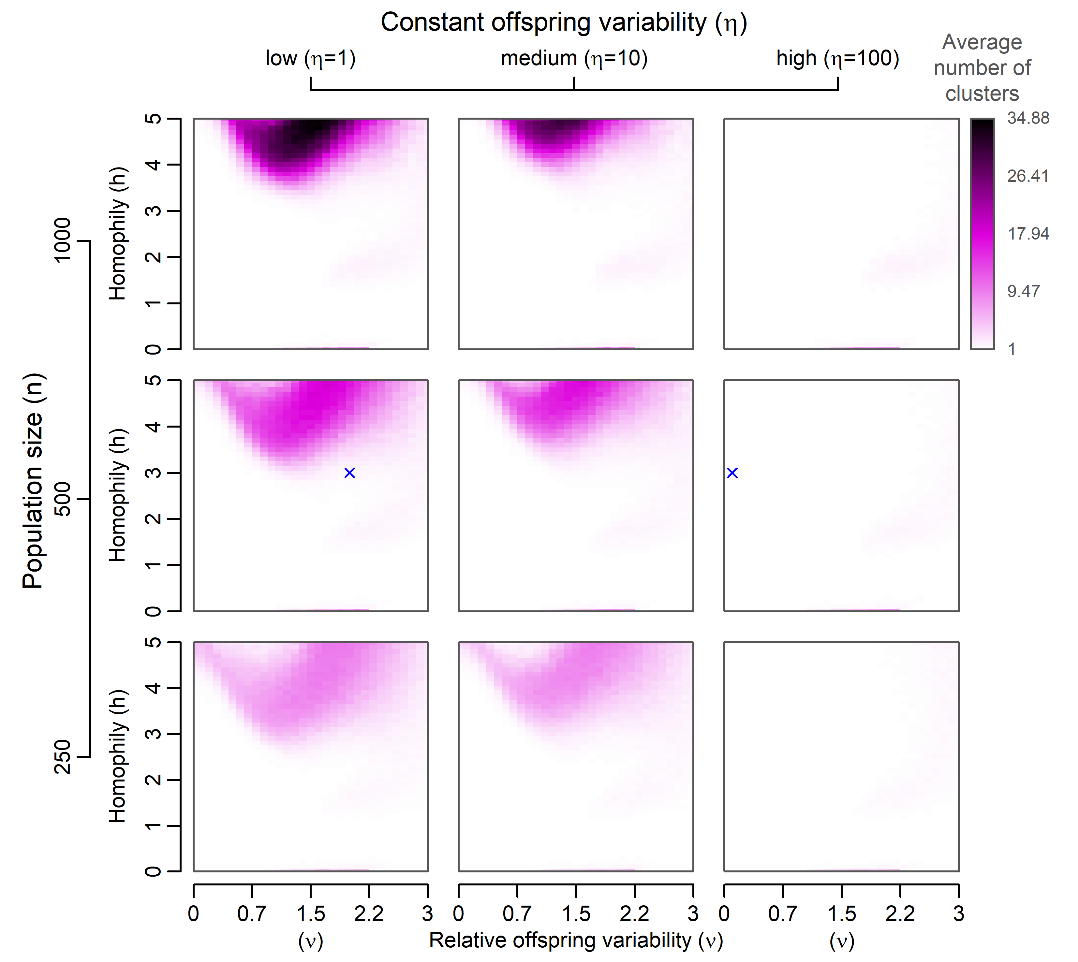
**

**Figure S20. A graphical summary of the tendency to form subcultures** **after 200 model generations in a system with additional normal noise**. The points in the 10-dimensional culture space were normally distributed across all dimensions at the beginning of each simulation run and 100 simulation runs were executed for each parameter combination. Blue crosses indicate examples available below.


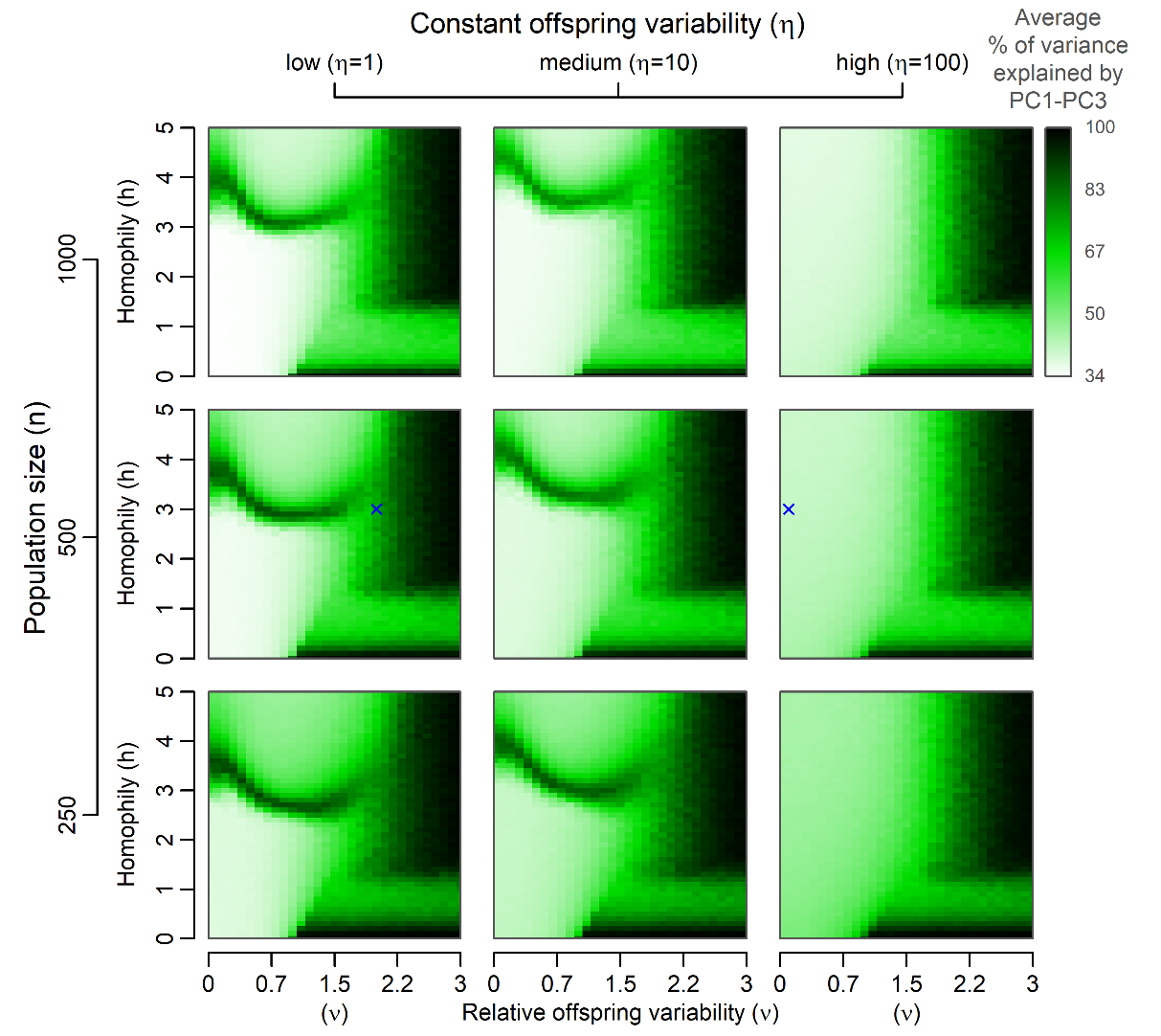


**Figure S21. A graphical summary of expected reduction in the number of effective dimensions after 200 model generations in a system an additional normal noise.** The same set of simulation runs as in Figure S20 was used to generate the image. PC1–PC3 stand for the first three principal components. Blue crosses indicate examples available below.


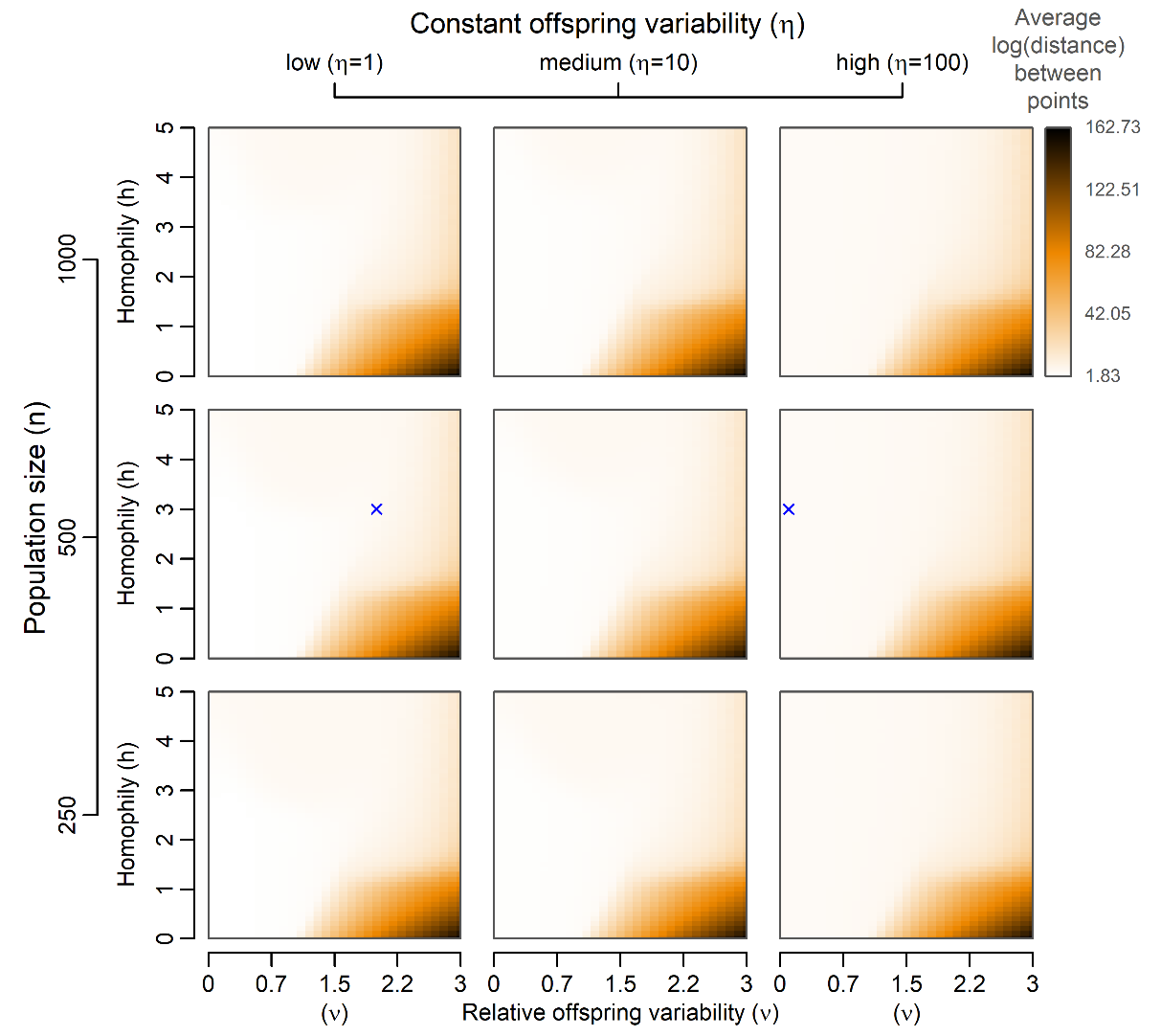


**Figure S22. A graphical summary of the expected average distance between agent positions in a culture space after 200 model generations in a system with additional random noise**. The same set of simulation runs as in Figure S20 was used to generate the image. Blue crosses indicate examples available below.


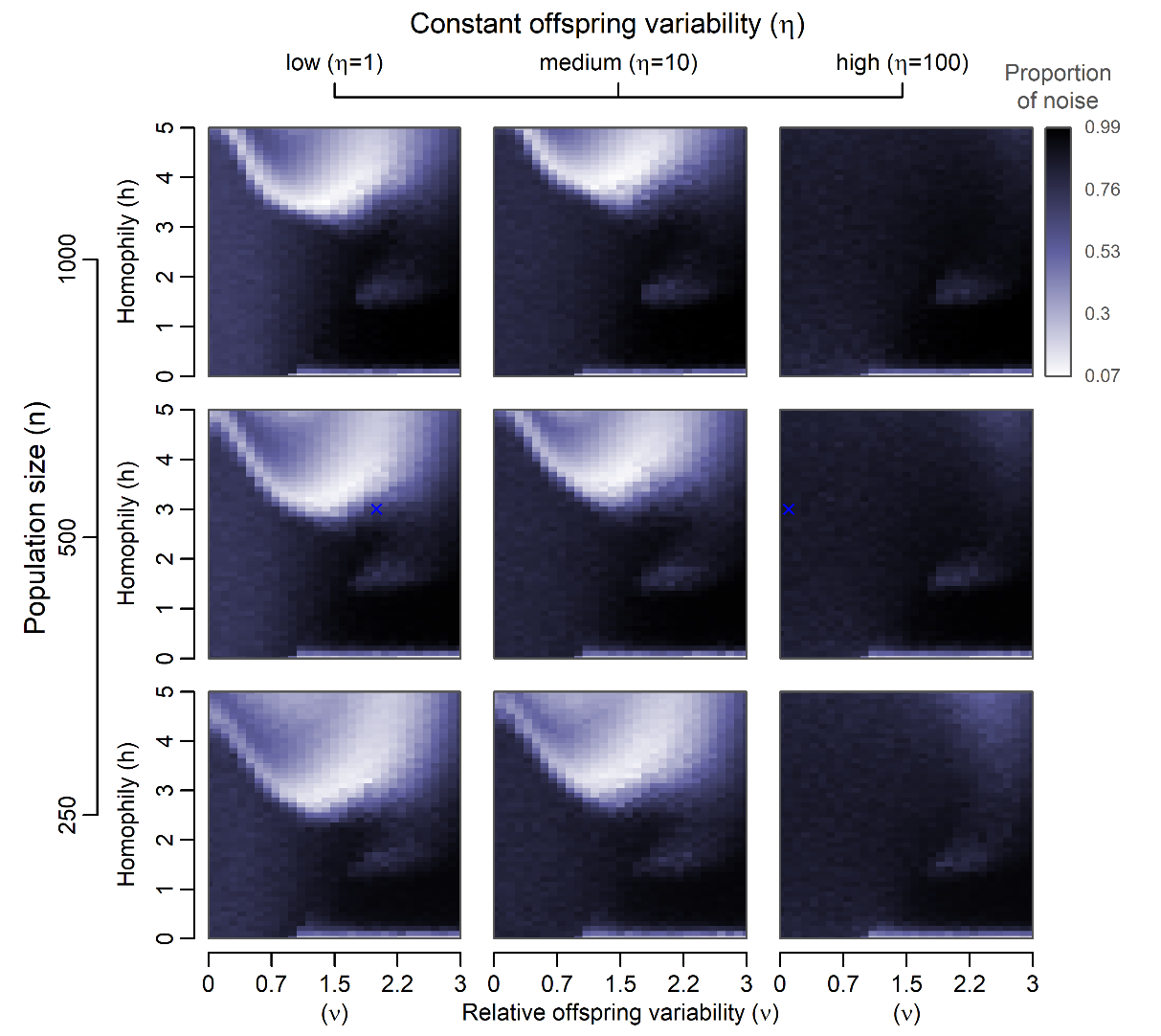


**Figure S23. A graphical summary of the proportion of noise (points that are not a part of any cluster)** after 200 model generations. The same set of simulation runs as in Figure 20 was used to generate the image. Blue crosses indicate examples available below.


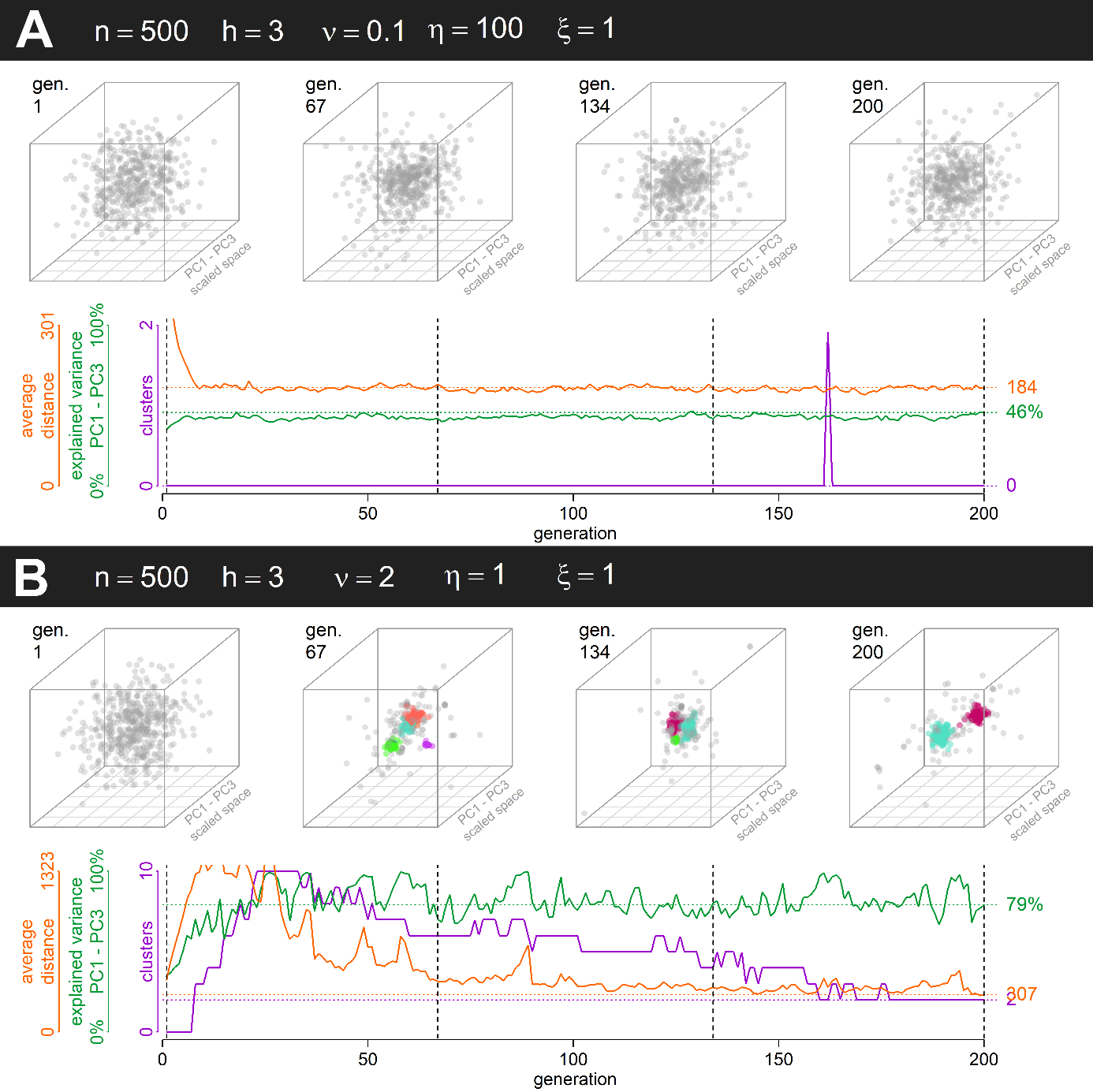


**Figure S24. Two simulation runs in a system with additional random noise, one with a strong influence of Galton-Pearson inheritance (A) and one with a strong influence of PVDI (B).** For detailed description see the caption of Figure 3 in the main article. Simulation run parameters were chosen to match those of Figure 3 Equivalents of Figures S5–S14 are not included for brevity, but can be easily obtained using the code at <https://doi.org/10.17605/osf.io/pvyhe> (See Supplementary animations S24A and S24B.)
