# Supplementary material for: How subcultures emerge
Source: Evol Hum Sci. 2023 Jul 12;5:e24. doi: 10.1017/ehs.2023.19 (PMC10426082; doi:10.1017/ehs.2023.19)

$n = 500 \quad h = 3 \quad \nu = 0.1 \quad \eta = 100$

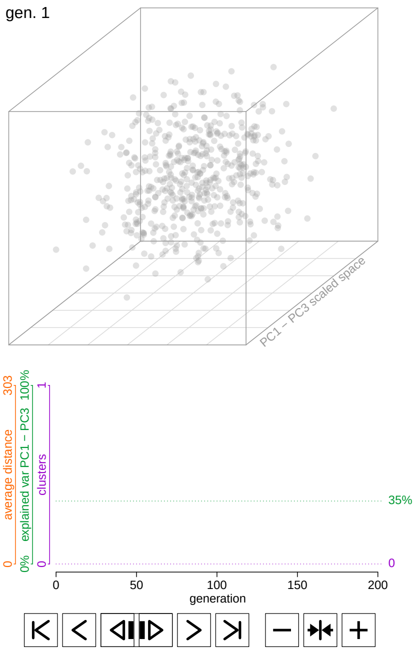

Supplement: Supplementary file 1 [file S2513843X23000191sup002.pdf › S2513843X23000191sup002.pdf]
